# Temporal and regional intestinal changes in permeability, tight junction, and cytokine gene expression following ovariectomy‐induced estrogen deficiency

**DOI:** 10.14814/phy2.13263

**Published:** 2017-05-03

**Authors:** Fraser L. Collins, Naiomy D. Rios‐Arce, Shelby Atkinson, Hayley Bierhalter, Daniel Schoenherr, Jason N. Bazil, Laura R. McCabe, Narayanan Parameswaran

**Affiliations:** ^1^ Department of PhysiologyMichigan State UniversityEast LansingMichigan; ^2^ Department of RadiologyMichigan State UniversityEast LansingMichigan; ^3^ Biomedical Imaging Research CentreMichigan State UniversityEast LansingMichigan

**Keywords:** Estrogen deficiency, inflammatory, intestine, menopause, permeability, tight junction

## Abstract

Estrogen deficiency that occurs during menopause is associated with wide‐ranging consequences, including effects on the gastrointestinal system. Although previous studies have implicated a role for estrogen in modulating colonic permeability and inflammatory gene expression, the kinetics of these changes following loss of estrogen and whether they are intestinal region specific are unknown. To test this, we performed sham or ovariectomy (OVX) surgery in BALB/c mice and examined permeability (in vivo and ex vivo) and gene expression changes in the duodenum, jejunum, ileum, and colon at 1, 4, and 8 weeks postsurgery. In vivo permeability, assessed by FITC‐dextran gavage and subsequent measures of serum levels, indicated that OVX significantly increased whole intestinal permeability 1 week postsurgery before returning to sham levels at 4 and 8 weeks. Permeability of individual intestinal sections, measured ex vivo by Ussing chambers, revealed specific regional and temporal responses to OVX, with the most dynamic changes exhibited by the ileum. Analysis of gene expression, by qPCR and by mathematical modeling, revealed an OVX‐specific effect with tight junction and inflammatory gene expression elevated and suppressed with both temporal and regional specificity. Furthermore, ileal and colonic expression of the tight junction protein occludin was found to be significantly correlated with expression of TNF *α* and IL‐1*β*. Together, our studies reveal previously unappreciated effects of estrogen deficiency in specific intestinal segments and further demonstrate temporal links between estrogen deficiency, inflammatory genes, and intestinal permeability.

## Introduction

Natural menopause, the permanent cessation of the menstrual cycle following the loss of ovarian follicular activity, is a process that typically occurs in women between 47 and 52 years of age (Davis et al. 2015). Menopause causes a rapid decline in circulating estrogen, which can have a detrimental effect on reproductive and nonreproductive tissues resulting in osteoporosis, urogenital atrophy, metabolic changes leading to muscle atrophy, lower basal metabolic rate and redistribution of body fat, increased risk of stroke, cognitive decline, and increased risk of depression (Burger et al. 1999; Khosla et al. 2011; Davis et al. 2012; Lisabeth and Bushnell 2012; Portman and Gass 2014; Weber et al. 2014). Menopause also increases serum levels of proinflammatory cytokines, including tumor necrosis factor *α* (TNF*α*), interleukin‐1*β* (IL‐1β), IL‐8, and IL‐6, as well as the osteoclastogenic cytokine receptor activator of nuclear factor‐*κ*B ligand (RANKL)(Jilka et al. 1992; Jabbar et al. 2011; Malutan et al. 2014). In addition, loss of estrogen is associated with intestinal changes that include decreased calcium absorption and changes in intestinal permeability which suggest that estrogen deficiency has direct effects on gastrointestinal homeostasis (Heaney et al. 1989; Li et al. 2016).

Ovariectomy (OVX), removal of the ovaries, is the gold standard murine model of estrogen deficiency (Jee and Yao 2001). While OVX causes a rapid loss of estrogen, similar to hysterectomy, the results produced using this model mirror the endpoints observed during menopause. Like menopause, ovariectomized mice exhibit a period of rapid bone loss (Jee and Yao 2001), urogenital atrophy (Ohlsson et al. 2014), and redistribution of body fat (Babaei et al. 2010; Davis et al. 2015). Thus, OVX provides a good model for understanding the effects of estrogen loss on organ function. The OVX model is routinely performed in adult animals, rather than an aged individual, as a plateau in the rate of bone growth has been reached. While aged animals would be more representative of human menopause, complicating factors including age have been shown to have a significant role in the regulation of the intestine and immune system, independent of estrogen loss (Pinchuk and Filipov 2008; Khosla et al. 2011). In rodent models of estrogen deficiency (ovariectomy, OVX), previous studies identified increased colonic permeability in both in vivo and ex vivo assays (Braniste et al. 2009; Li et al. 2016). These changes were associated with decreased expression of tight junction proteins occludin, members of the claudin family, and junction‐associated adhesion molecule (JAM) 3 in the colon (Braniste et al. 2009; Li et al. 2016). Furthermore, estrogen receptor‐β (ER‐β) knockout mice exhibit decreased cell adhesion molecules and a disrupted tight junction in the colon as well as abnormal colon architecture (Wada‐Hiraike et al. 2006). Additionally, colonic inflammation is associated with decreased ER‐β expression and increased colonic permeability (Looijer‐van Langen et al. 2011). These studies suggest that estrogen signaling is important in colonic homeostasis; however, the role of estrogen signaling or the consequence of estrogen deficiency on other intestinal segments is unknown.

Estrogen is also a critical regulator of intestinal immune cell functions, loss of which has been shown to increase expression of proinflammatory cytokines in immune cells isolated from the small intestine (Li et al. 2016). Estrogen receptors ER‐*α* and ER‐*β* are expressed to varying degrees in the gut‐associated immune cells (Phiel et al. 2005; Kovats 2015). Signaling through ER‐*α* in CD4^+^ T cells has been shown to have anti‐inflammatory properties inhibiting Th1/Th17 priming (Lélu et al. 2011). Correspondingly, ER‐α‐deficient macrophages and dendritic cells express higher levels of TNFα in response to lipopolysaccharide (Lambert et al. 2004). Together, these studies indicate that estrogen deficiency could lead to local intestinal changes in inflammatory cytokine expression akin to those seen in the blood and bone marrow.

Although previous studies suggest that estrogen deficiency can modulate intestinal permeability and inflammatory gene expression, the development of these changes subsequent to estrogen depletion, and whether the changes occur in the various regions of the intestine or are localized to specific intestinal segments, is not known. Here, we define the kinetics of expression of tight junction and inflammatory genes in the different segments of the small and large intestine following estrogen deficiency. We find that changes in in vivo and ex vivo permeability are time and region dependent, and that expression of tight junction and inflammatory genes are modulated in both temporal and regional specificity. Using modeling approaches, we also provide models of changes in these genes following estrogen deficiency. These findings reveal that care should be taken when extrapolating data from single time‐point measurements. Furthermore, they demonstrate complex and multidimensional effects on the intestine, consequent to estrogen deficiency while providing targets and regions for future investigation.

## Methods

### Ethical approval

All animal procedures were approved by the Michigan State University Institutional Animal Care and Use Committee and conformed to NIH guidelines.

### Animals and experimental design

Female BALB/c mice 11 weeks of age were obtained from The Jackson Laboratory (Bar Harbour, Maine). Mice were allowed to acclimate to animal facility for 1 week prior to start of experiment. Animals were randomly split into two groups: sham control or OVX. For sham and OVX surgeries, mice were anesthetized with isofluorane and a 2 cm lower mid‐dorsal incision was made extending through the skin and muscle layers. Ovaries were isolated in both sham and OVX groups; ovaries were removed from the OVX cohort and incision sites closed using surgical staples in both sham and OVX mice. Mice were given Teklad 2019 chow (Madison, WI) and water ad libitum and were maintained on a 12‐h light/dark cycle. Mice were sacrificed at 1, 4, and 8 weeks postsurgery.

### In vivo permeability

In vivo intestinal permeability was evaluated by measuring paracellular permeability to 4 kDa fluorescein isothiocyanate (FITC)‐dextran as described previously (Laukoetter et al. 2007; Lee et al. 2015; Li et al. 2016). Briefly, mice were administered 300 mg/kg FITC‐dextran in a total volume of 150 *μ*L by oral gavage. Serum was obtained 4 h after administration by cardiac puncture under isoflurane anesthesia and fluorescence intensity measured with a microplate reader (excitation, 485 nm; emission, 530 nm; Tecan, Morrisville, NC). FITC‐dextran concentrations were determined using a standard curve and normalized against time.

### Ex vivo Ussing chamber intestinal permeability

Mice were sacrificed at designated time points and proximal segments of the duodenum, jejunum, and ileum and distal colon were removed. Sections were mounted in Lucite chambers and placed in Ussing chambers (Physiologic Instruments, San Diego, CA) exposing mucosal and serosal surfaces to oxygenated (95% O_2_, 5% CO_2_) Krebs bicarbonate ringer buffer (Sigma, St. Louis, MO). Intestinal sections were not stripped of underlying muscle. Buffer was maintained at 37°C by a heated water jacket and samples were allowed to equilibrate for 30 min. Transepithelial conductance (*G*_t_) was measured by clamping the voltage and recording the change in the short‐circuit current (*I*_sc_) following a pulsed command voltage every 20 sec.

For measurements of tissue flux, 4 kDa FITC‐dextran (2.2 mg/mL final concentration) was added to the mucosal chamber; 10 kDa rhodamine B isothiocyanate (RITC)‐dextran (0.55 mg/mL final concentration) was also added to the mucosal chamber and used as a control for tissue integrity. Serosal chamber samples were taken at 0 and 60 min, and fluorescence intensity determined (FITC excitation, 485 nm; emission, 530 nm; RITC excitation, 595 nm; emission, 615 nm; Tecan). FITC‐dextran/RITC‐dextran concentrations were determined using a standard curve and FITC‐dextran flux in OVX mice normalized against sham mice and reported as fold change.

### Intestine RNA analysis

Sections from the proximal duodenum, jejunum, and ileum and the distal colon were frozen and crushed under liquid nitrogen conditions with a Bessman Tissue Pulverizer (Spectrum Laboratories, Rancho Dominguez, CA). RNA was isolated from frozen samples using TriReagent (Molecular Research Center, Cincinnati, OH) and integrity assessed by formaldehyde‐agarose gel electrophoresis. cDNA was synthesized by reverse transcription using Superscript II Reverse Transcriptase Kit and oligo dT(12–18) primers (Invitrogen, Carlsbad, CA). cDNA was amplified by quantitative qPCR with iQ SYBR Green Supermix (BioRad, Hercules, CA), and gene‐specific primers (synthesized by Integrated DNA Technologies, Coralville, IA; Table 1). Hypoxanthine guanine phosphoribosyl transferase (HPRT) mRNA levels were used as an internal control. Real‐time PCR was carried out for 40 cycles using the iCycler (Bio‐Rad) and data evaluated using the iCycler software. Each cycle consisted of 95°C for 15 sec, 60°C for 30 sec, and 72°C for 30 sec. Negative controls included primers without cDNA. Expression of all genes was normalized to 1 week sham duodenum.

**Table 1:** qPCR primers

Gene	Forward primer (5′–3′)	Reverse primer (5′–3′)
*HPRT*	AAGCCTAAGATG AGCGCAAG	TTACTAGGCAGATGGCCACA
*TNFα*	AAGGGAGAGTGGTCAGGTTGCC	CCTCAGGGAAGAGTCTGGAAAGG
*TGFβ1*	GCAACAATTCCTGGCGTTACC	CCCTGTATTCCGTCTCCTTGGT
*IFNγ*	GGCTGTCCCTGAAAGAAAGC	GAGCGAGTTATTTGTCATTCGG
*IL‐1β*	TCCCCGTCCCTATCGACAAAC	GCGGTGATGTGGCATTTTCTG
*IL‐17A*	TGAGCTTCCCAGATCACAGA	TCCAGAAGGCCCTCAGACTA
*IL‐10*	GGTTGCCAAGCCTTATCGGA	ACCTGCTCCACTGCCTTGCT
*IL‐22*	TCGCCTTGATCTCTCCACTC	GCTCAGCTCCTGTCACATCA
*IL‐23p19*	AGGCTGCCCTTTGAAGATGT	CCAGCGGGACATATGAATCT
*Occludin*	GCTCAGGGAATATCCACCTAT	CACAAAGTTTTAACTTCCCAGACG
*Jam3*	ATGTACCACTGGGTTTCGGT	CTGCCTGACTTCTTCCTGCT
*ZO‐1*	AGGACACCAAAGCATGTGAG	GGCATTCCTGCTGGTTACA
*Claudin 1*	TCTACGAGGGACTGTGGATG	TCAGATTCAGCAAGGAGTCG
*Claudin 2*	GGCTGTTAGGCACATCCAT	TGGCACCAACATAGGAACTC
*Claudin 3*	AAGCCGAATGGACAAAGAA	CTGGCAAGTAGCTGCAGTG
*Claudin 4*	CGCTACTCTTGCCATTACG	ACTCAGCACACCATGACTTG
*Claudin 5*	GTGGAACGCTCAGATTTCAT	TGGACATTAAGGCAGCATCT
*Claudin 8*	GCCGGAATCATCTTCTTCAT	CATCCACCAGTGGGTTGTAG

### Statistical analysis

Significant outliers were removed using the Grubbs' test for outliers. The data were transformed to fold change relative to week 1 expression levels in the duodenum to allow determination of relative gene expression across the length of the intestine. Hierarchical clustering was performed using Euclidean pairwise distance metrics for temporal expression and correlation pairwise distance metrics for gene expression. Distance metrics for generating clusters were computed using the furthest distance between each cluster. Dendrograms were then computed from the clustered data for both gene expression and time points. Heat maps were designed to cluster together tight junctions/inflammatory cytokines that have similar changes in gene expression. Furthermore, the heat maps were designed to cluster time points based on similarity; therefore, depending on the outcomes, the different heat maps have different timings. To compute the OVX effect, the *P* values from the paired *t*‐tests comparing control versus ovariectomy data were transformed to log10 values, and nonparametric, smoothing splines were computed from the data.

## Results

### Effect of sham and OVX on general body parameters

In humans, loss of ovarian function during menopause is associated with a rapid increase in adipose tissue mass, redistribution of fat to the abdomen, and decreased uterine size (Merz et al. 1996; Davis et al. 2012). We examined these parameters in the murine OVX model of estrogen deficiency at 1, 4, and 8 weeks following surgery (Table 2). Although at 1 week postsurgery, there was no significant difference in body weight between OVX and sham groups, at 4 and 8 weeks postsurgery, body weights were significantly higher in the OVX (22.4 ± 0.6 g and 21.6 ± 0.4 g, respectively) compared to the sham cohort (20.7 ± 0.4 g and 20.2 ± 0.3 g, respectively). Inguinal and retroperitoneal fat pad weights in OVX mice decreased at 1 week, but increased at 8 weeks postsurgery. Specifically, at 1 week postsurgery in OVX versus sham mice the inguinal fat mass displayed a 42% decrease (104 ± 15 mg vs. 177 ± 13 mg, respectively) and the retroperitoneal fat mass displayed a 58% decreased (14 ± 3 mg vs. 33 ± 3 mg, respectively). By 4 weeks postsurgery, there were no differences in fat pad weights. In contrast, by 8 weeks postsurgery, the OVX inguinal fat mass was increased by 27% (150 ± 12 mg vs. 118 ± 8 mg, respectively) and the retroperitoneal fat mass was increased by 32% (65 ± 4 mg vs. 47 ± 4 mg, respectively) over sham. These changes in fat mass were present even when corrected for body weight, with the exception of the inguinal fat at 8 weeks (data not shown). As expected, uterine weights were significantly decreased in OVX animals (compared to sham) as early as 1 week postsurgery, which persisted throughout all the time points tested.

**Table 2:** General body parameters

	1 Week		4 Weeks		8 Weeks	
	Sham	OVX	Sham	OVX	Sham	OVX
Weight (g)	20.5 ± 0.3	20.3 ± 0.4	20.7 ± 0.4	**22.4 ± 0.6** **	20.2 ± 0.3	**21.6 ± 0.4** **
Inguinal fat (mg)	177 ± 13	**104 ± 15** **	144 ± 11	133 ± 10	118 ± 8	**150 ± 12** *
Retroperitoneal fat (mg)	32 ± 3	**14 ± 3** *	18 ± 2	20 ± 2	47 ± 4	**65 ± 4** **
Spleen (mg)	83 ± 7	102 ± 7	102 ± 4	103 ± 5	109 ± 4	105 ± 3
Uterus (mg)	99 ± 6	**32 ± 3** **	103 ± 9	**24 ± 2** **	133 ± 12	**40 ± 5** **

*P < 0.05, **P < 0.01 significant to relevant control sham.

### Temporal changes in intestinal permeability following OVX

**Figure 1:**
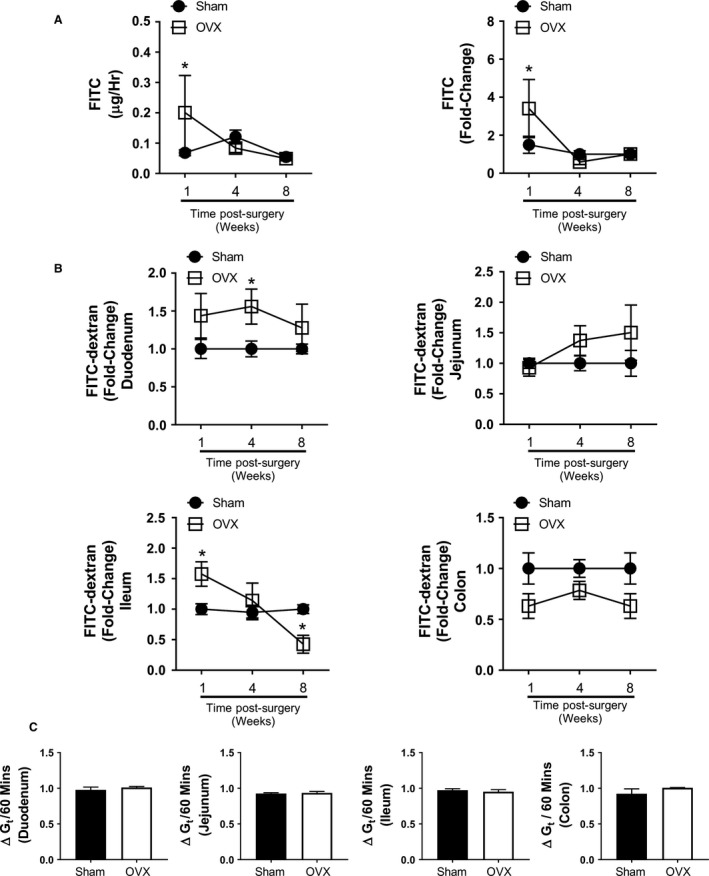
Estrogen deficiency modulates in vivo and ex vivo regional intestinal permeability. Female BALB/c mice (12 weeks) underwent either sham or ovariectomy (OVX) surgery. Mice were gavaged 1 week (*n* = 5), 4 weeks (*n* = 9), and 8 weeks (*n* = 10) postsurgery with FITC‐dextran and serum obtained 4 h later to measure (A) in vivo intestinal permeability. Estrogen deficiency significantly increased intestinal permeability 1 week postsurgery in the OVX mice compared to the sham (*P *<* *0.05). No significant difference was observed at the 4 week and 8 week time points. Intestinal sections were removed at experimental endpoints and (B) regional permeability determined ex vivo using Ussing chambers. Mean flux of FITC‐dextran observed across sham intestinal sections: duodenum, 8.1; jejunum, 13.2; ileum, 17.9; and colon, 4.0 *μ*g/h/cm^2^. Significantly increased permeability was detected in the OVX ileum at 1 week (*P *<* *0.05) and the OVX duodenum at 4 weeks (*P *<* *0.05). A significant decrease in permeability was identified in the OVX ileum 8 weeks postsurgery (*P *<* *0.05), while permeability in the OVX colon trended lower across the time course. Representative results for the (C) fold change in intestinal section transepithelial conductance (*G*_t_) for the duration of FITC‐dextran measurement in the Ussing chambers. Mean baseline sham *G*_t_ recorded as: duodenum, 4.02; jejunum, 4.8; ileum, 5.9; and colon, 4.8 mS/cm^2^. Statistical analysis performed by multiple *t*‐test corrected for multiple comparisons by the Holm–Šídák method.

To systematically establish the onset of changes and long‐term effects of OVX on intestinal barrier function, we examined in vivo intestinal permeability (as a measure of overall gut permeability) as well as region‐specific ex vivo permeability of duodenum, jejunum, ileum, and colon at 1, 4, and 8 weeks following surgery. At 1 week postsurgery, a significant increase in in vivo intestinal permeability was observed in the OVX cohort compared to the sham controls (Fig. 1A). Interestingly, at 4 and 8 weeks postsurgery, no significant differences in in vivo intestinal permeability were observed between sham and OVX mice, suggesting that estrogen deficiency affects intestinal permeability only at early onset, and that by 4 weeks permeability is restored to sham levels. Analysis of region‐specific permeability, by assaying individual gut sections ex vivo, revealed both temporal and regional differences (Fig. 1b). One week postsurgery, only the ileum exhibited a significant increase in permeability in OVX compared to sham. Other segments, at this point, did not show any marked differences except for a modest decrease in the OVX colon (*P *=* *0.08). Interestingly, 4 weeks after surgery, permeability was significantly increased only in the OVX duodenum (*P *<* *0.01) with no significant differences observed in the jejunum, ileum, or colon. By 8 weeks postsurgery, there was a significant decrease in permeability in the OVX compared to sham ileum, while other sections did not have any differences in permeability. ANOVA comparing the OVX and sham intestinal sections revealed that OVX had a significant effect on duodenal (*P *<* *0.05) and colonic permeability (*P *<* *0.01) across the time course. Transepithelial conductance (*G*_t_) was recorded as a measure of tissue integrity, no significant change in *G*_t_ was observed in any of the segments during the time period of permeability measurements (Fig. 1C). Together, these data suggest that changes in ileal permeability early after OVX surgery is likely responsible for the observed increase in in vivo overall gut permeability in the estrogen‐deficient mice. These results also demonstrate that the overall changes in intestinal permeability are not only time‐dependent following estrogen deficiency, they are dependent on specific intestinal segments.

### Estrogen deficiency alters tight junction protein gene expression

**Figure 2:**
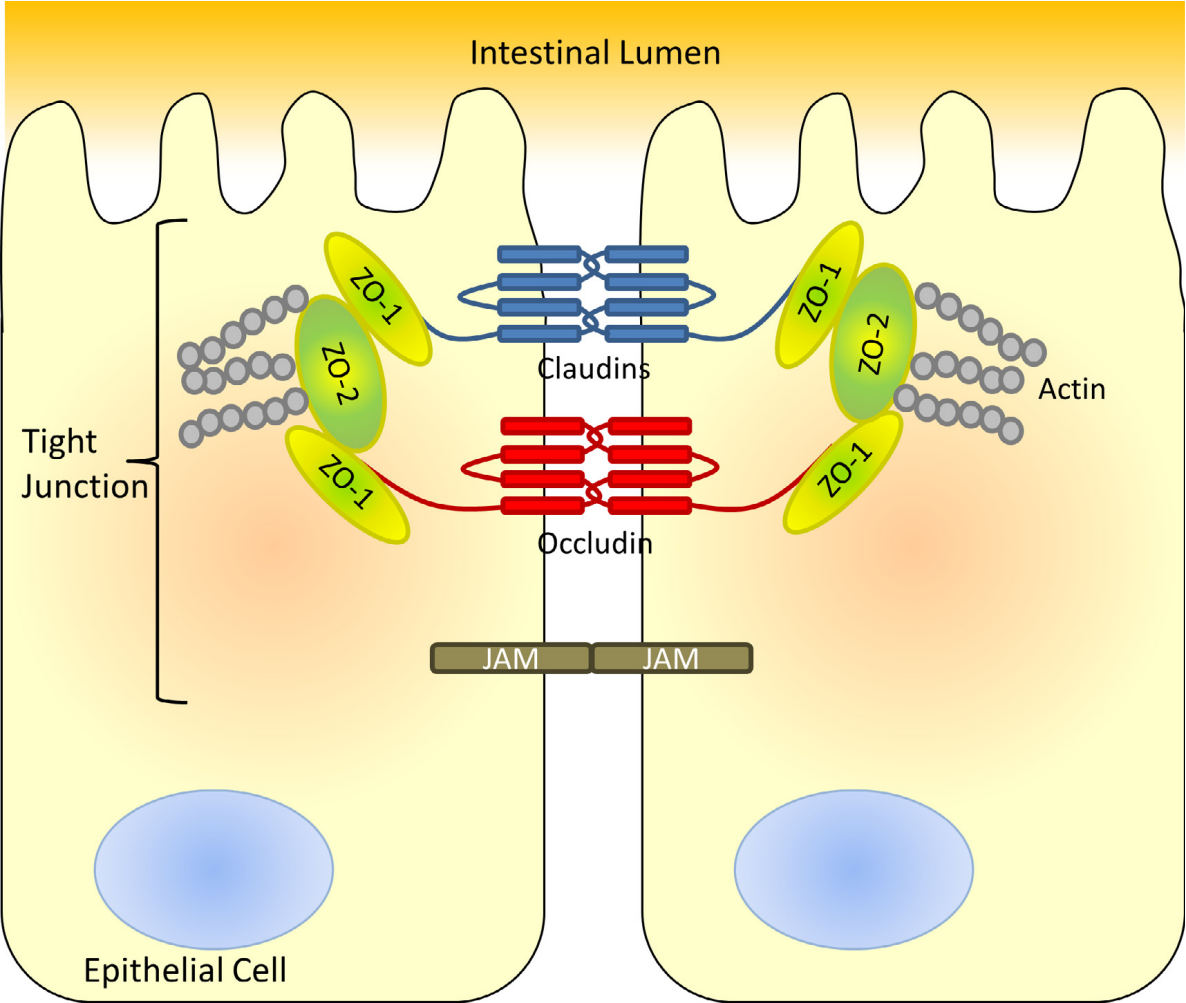
Intestinal tight junction proteins. Schematic representation of intestinal epithelial cell tight junction proteins and their location. Modified from Neunlist et al. (2013).

**Figure 3:**
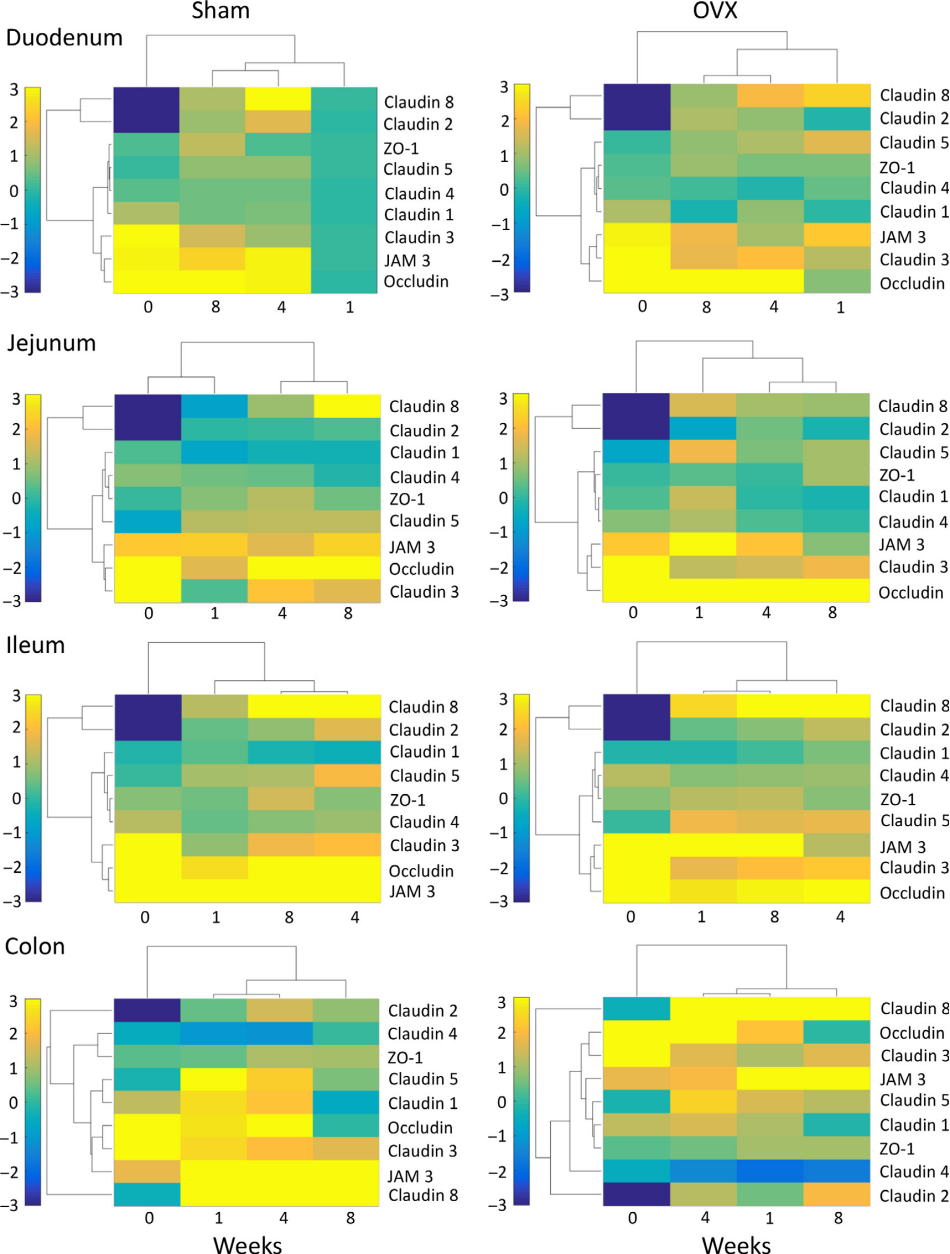
Changes in tight junction gene expression following sham and OVX surgeries. Female BALB/c mice (12 weeks) underwent either sham or ovariectomy (OVX) surgery. Intestinal regions were isolated and tight junction gene expression analyzed by qPCR. Heat maps were designed to cluster together tight junction proteins that have similar changes in gene expression, relative to week 1 expression in the duodenum of the sham control, and cluster time points based on similarity. *N* = 5–11.

Intestinal paracellular permeability and barrier function are controlled through the expression of numerous tight junction proteins including members of the claudin family, occludin, the junctional adhesion molecules (JAM) family, and zonula occludens (ZO) (Fig. 2; Suzuki 2013). Therefore, we measured gene expression of tight junction proteins in the various segments of the intestine to determine whether estrogen deficiency alters intestinal permeability through modulation of the tight junction. Tight junction gene profiles were first organized using hierarchical clustering. Figure 3 shows the clustered expression levels, relative to week 1 expression in the duodenum of the sham control, for the nine tight junction protein genes and the four time points, intact (0) to 8 weeks postsurgery (8), in each of the gut sections for sham and OVX mice. In response to surgery, changes in tight junction gene expression were observed across all regions of the intestine. Large increases in relative gene expression of occludin, claudin‐3, and JAM3 were observed in both the sham and OVX in all regions relative to the sham 1 week duodenum. Modest changes in gene expression were detected for the clusters: claudin‐1, claudin‐4, claudin‐5, and ZO‐1; and claudin‐2 and claudin‐8 in the duodenum, jejunum, and ileum. However, in the colon, differing gene clustering patterns and expression were observed in relation to the rest of the intestine. Analysis of temporal clustering identified differences between the intestinal regions and between sham and OVX. However, across all intestinal regions, in both sham and OVX mice, the intact time point was furthest removed except for the sham jejunum. The heat maps reveal that both surgery and OVX have a profound effect on tight junction gene expression throughout the intestine and that these effects are long‐lasting. Furthermore, they identify that certain genes are clustered together in the small intestine, whereas the large intestine exhibits a differing response.

**Figure 4:**
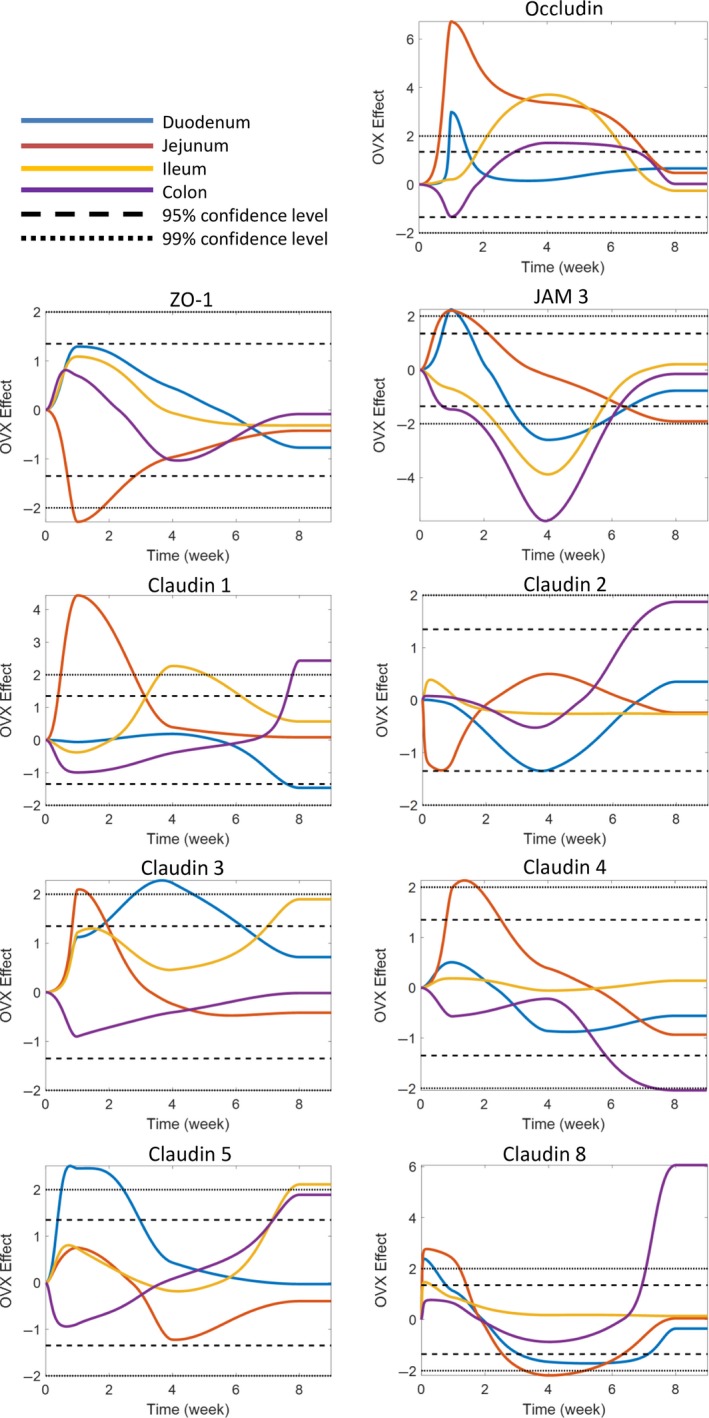
Effect of OVX on tight junction gene expression. Female BALB/c mice (12 weeks) underwent either sham or ovariectomy (OVX) surgery. Intestinal regions were isolated and tight junction gene expression analyzed by qPCR. Statistical testing was used to identify the specific effect of OVX on tight junction gene expression. Graphs represent regional and temporal changes (upregulated and downregulated) in OVX gene expression compared to the sham control. Changes greater than 95% confidence level was determined to be significant. *N* = 5–11.

To identify the specific effect of OVX over sham surgery on tight junction gene expression, statistical testing was employed (Fig. 4). The results were then related back to the hierarchical analysis and qPCR analysis to determine whether OVX resulted in an upregulation or suppression of gene expression over the sham control. In the duodenum, OVX affected expression of occludin, JAM3, and claudin‐5, which rose significantly early on in the time course (1 week; CI > 99%), while levels of claudin‐3 (CI > 99%) increased at 4 weeks. In contrast, claudin‐8 (CI > 95%) was significantly downregulated 4 weeks postsurgery. Interestingly, 8 weeks postsurgery no significant differences in gene expression was observed between the sham and OVX duodenum with the exception of claudin‐1, which was significantly lower in the OVX cohort (CI > 95%).

A similar pattern in gene expression was observed in the OVX jejunum. Occludin and JAM3 were again significantly (CI > 99%) upregulated at 1 week. In addition, claudin‐1, claudin‐3, claudin‐8 (CI > 99%), and claudin‐4 (CI > 95%) were also observed to be elevated, while ZO‐1 (CI > 99%) was suppressed. Four weeks postsurgery, occludin was still significantly elevated in the OVX cohort in contrast to claudin‐8 expression which was significantly reduced compared to the sham. As with the duodenum, the majority of jejunal tight junction gene expression was comparable between sham and OVX at 8 weeks; however, expression of JAM3 was significantly reduced (CI > 95%) in the OVX mice.

Ileal tight junction gene expression was not affected 1 week following OVX, in contrast to the changes seen in the duodenum and jejunum. However, by 4 weeks, occludin and claudin‐1 (CI > 99%) were elevated in the ileum of OVX mice, while claudin‐3 (CI > 95%) and claudin‐5 (CI > 99%) expression increased at 8 weeks. JAM3 expression was reduced at 4 weeks (CI > 99%).

Analysis of the colonic tight junction gene expression identified divergent expression compared to the other intestinal sections. At 1 week, OVX resulted in a significant reduction in gene expression of occludin (CI > 95%) and JAM3 (CI > 99%). However, at 4 weeks expression of occludin was significantly increased (CI > 95%), while JAM3 remained decreased (CI > 99%). Eight weeks post‐OVX, expression of claudin‐1, claudin‐8 (CI >99%), claudin‐2, and claudin‐5 (CI > 95%) increased, whereas claudin‐4 was significantly lowered (CI > 99%).

The expression analysis of these various tight junction genes reveals that OVX has an effect over and above that of surgery alone demonstrating that estrogen deficiency profoundly affects tight junction genes in both a temporal and intestinal segment‐specific manner. Furthermore, the data also suggest that changes in the expression of some of these tight junction genes track with changes in permeability (especially ileum), while in some segments (especially colon), the relationship between the expression of these tight junction genes and permeability is not as clear.

### Changes in intestinal pro‐ and anti‐inflammatory cytokine expression following estrogen deficiency

**Figure 5:**
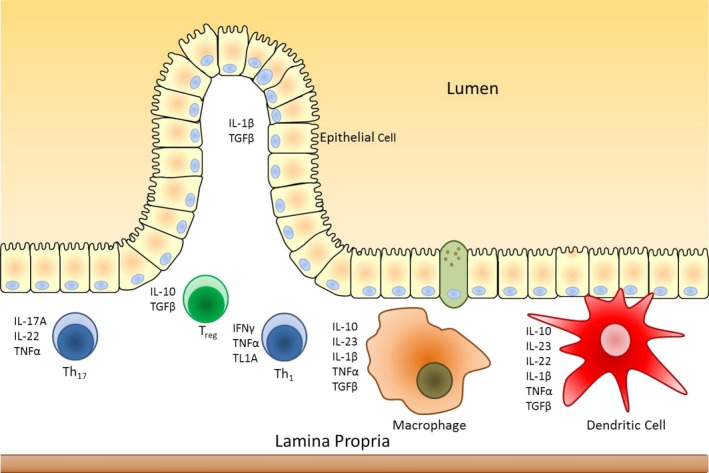
The intestinal immune system. Schematic representation of the intestinal immune system and cytokines produced. Modified from Neurath (2014).

Cytokines are known to play an important role in the regulation of tight junction protein expression under pathological conditions (Lee 2015). While estrogen deficiency increases expression of proinflammatory cytokines in the blood and bone marrow, the local effect on the regions of gut remains less clear. Therefore, we analyzed regional intestinal inflammatory gene expression to determine whether estrogen deficiency‐induced changes in pro‐ and anti‐inflammatory genes can be correlated with observed changes in temporal and regional tight junction gene expression and intestinal permeability (Fig. 5).

**Figure 6:**
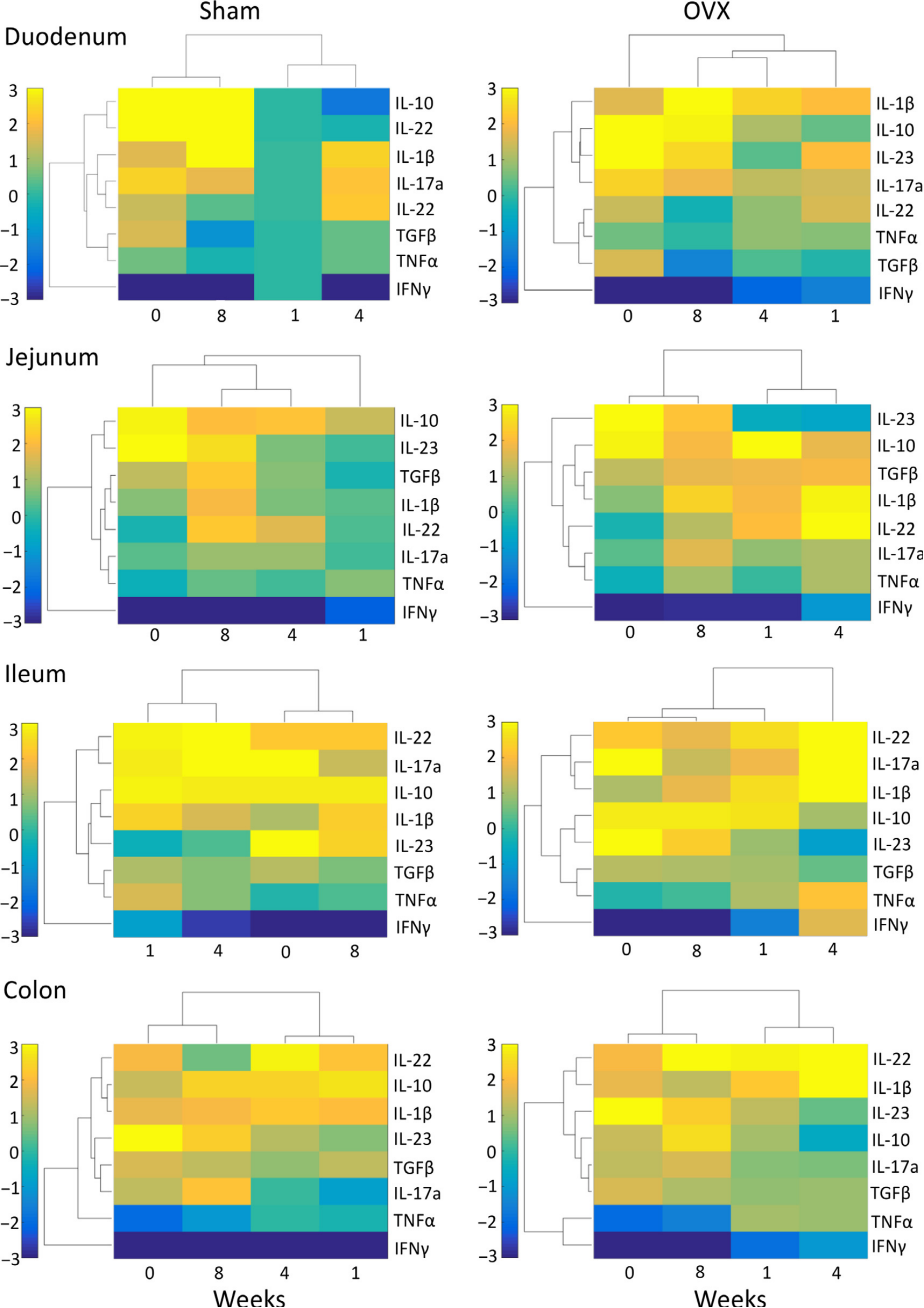
Changes in cytokine gene expression following sham and OVX surgeries. Female BALB/c mice (12 weeks) underwent either sham or ovariectomy (OVX) surgery. Intestinal regions were isolated and pro‐ and anti‐inflammatory cytokine gene expression analyzed by qPCR. Heat maps were designed to cluster together inflammatory cytokines that have similar changes in gene expression, relative to week 1 expression in the duodenum of the sham control, and cluster time points based on similarity. *N* = 5–11.

As with the tight junction proteins, inflammatory cytokine gene expression and time points were organized via hierarchical clustering for the different intestinal segments (duodenum, jejunum, ileum, and colon) in the sham and OVX mice (Fig. 6). In some cases, cytokine gene expression levels were up‐ and downregulated throughout the intestine in both sham and OVX, suggesting that surgery itself can influence intestinal cytokine expression levels. These changes, however, were region specific as no consistent clustering of genes between sections was observed, except for IFN*γ* which was separate from the other cytokines. Members of the IL‐10 family (IL‐10 and IL‐22) tended to be increased in the ileum and the colon throughout the experimental time course, with some exceptions observed in the OVX mice. Interestingly, in relation to other proinflammatory cytokines such as IL‐17A and IL‐1*β*, TNF*α*, expression only exhibited modest changes throughout the intestine. Temporal clustering was identified to be region and condition specific with no discernible pattern observed across the whole intestine. However, in the sham duodenum, ileum, and colon and in the OVX jejunum, ileum, and colon, the cytokine gene expression profiles in intact (0) and 8 weeks were observed to cluster together, suggesting that inflammation returns to presurgery levels by 8 weeks. Comparable to the tight junction analysis, both surgery and OVX resulted in both regional and temporal changes in intestinal cytokine gene expression. In contrast to the tight junctions, however, no discernible pattern in gene clustering was observed between the different intestinal sections indicating that the inflammatory response to surgery and OVX is region specific.

**Figure 7:**
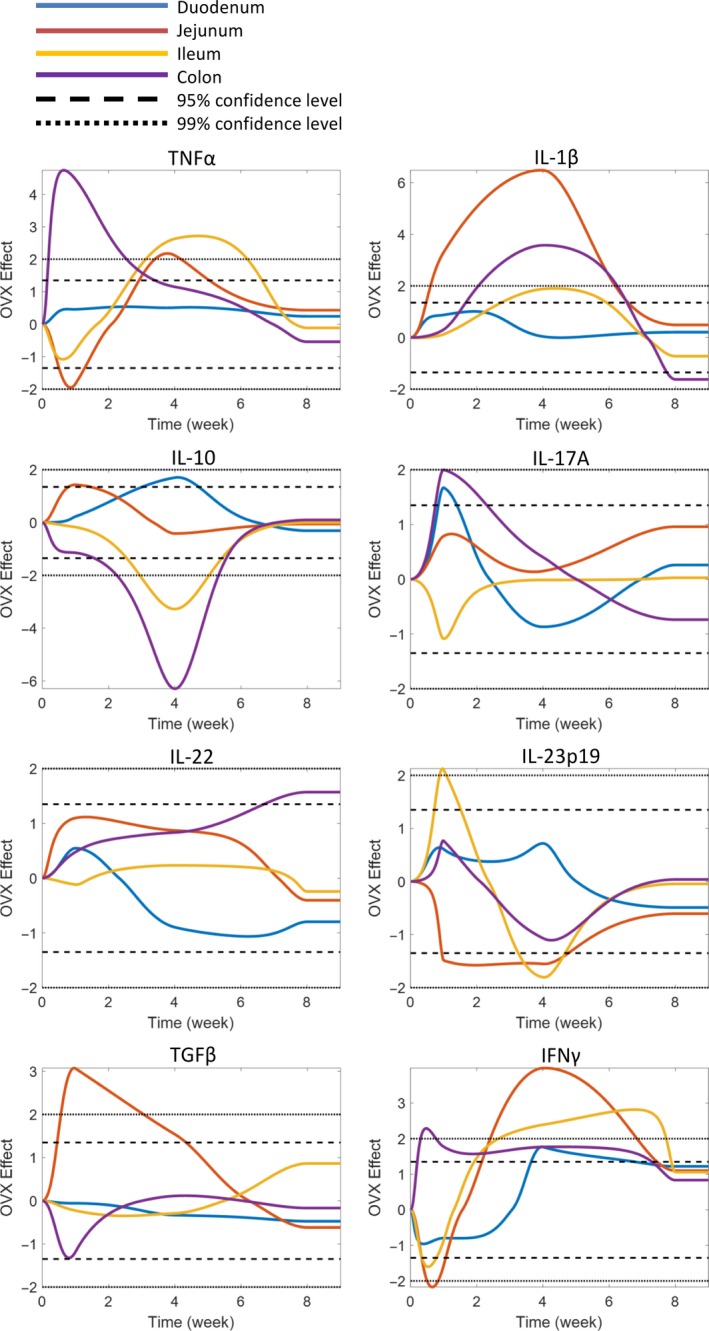
Effect of OVX on intestinal pro‐ and anti‐inflammatory cytokine gene expression. Female BALB/c mice (12 weeks) underwent either sham or ovariectomy (OVX) surgery. Intestinal regions were isolated and pro‐ and anti‐inflammatory cytokine gene expression analyzed by qPCR. Statistical testing was used to identify the specific effect of OVX on pro‐ and anti‐inflammatory cytokine gene expression. Graphs represent regional and temporal changes (upregulated and downregulated) in OVX gene expression compared to the sham control. Changes greater than 95% confidence level was determined to be significant. *N* = 5–11.

We next sought to distinguish the effect of OVX on intestinal cytokine gene expression from that of sham surgery by statistical analysis (Fig. 7), with the results related back to the hierarchical analysis and qPCR analysis and are noted below in detail for both the small and large intestine.

Specific time‐ and site‐dependent cytokine changes were observed in the small intestine. In the duodenum, at 1 week post‐OVX, expression of IL‐17A was significantly elevated (CI > 95%) compared to the sham. Levels of IL‐10 and IFN*γ* (CI > 95%) were observed to be elevated at the 4‐week time point. Eight weeks postsurgery, no differences in gene expression were observed between the sham and the OVX. Analysis of the jejunum identified even more significant changes in cytokine gene expression. IL‐1*β*, TGF*β* (CI > 99%), and IL‐10 (CI > 95%) were upregulated, while TNF*α* and IL‐23p19 were downregulated 1 week post‐OVX surgery. At 4 weeks postsurgery, IL‐1*β* (CI > 99%) and TGF*β* (CI > 95%) expression were still elevated in addition to increased TNF*α* and IFN*γ* (CI > 99%). Expression of IL‐23p19 was suppressed (CI > 95%). Similar to the duodenum, gene expression between the sham and the OVX was comparable at the 8 week time point. In contrast to the proximal small intestine, the ileum displayed only minor OVX‐induced changes in gene expression after 1 week, except for a significant increase in IL‐23p19 expression (CI >99%). However, by the 4 week time point, expression of TNF*α*, IFN*γ* (CI >99%), and IL‐1*β* (CI >95%) were significantly increased, whereas IL‐10 (CI >99%) and IL‐23p19 (CI >95%) were decreased. Again, no difference was observed at 8 weeks except for decreased IL‐23p19 expression (CI > 99%), comparable with the other sections of the small intestine, supporting the concept that with extended time the estrogen deficiency‐induced inflammatory processes likely return to control levels in the small intestine.

In the large intestine, significant OVX‐induced changes in gene expression were revealed at 1 week for TNF*α* (CI > 99%), IL‐17A, and IFN*γ* (CI > 95%). Four weeks postsurgery, IFN*γ* was still upregulated (CI > 95%) in addition to IL‐1*β* (CI > 99%). Expression of IL‐10 (CI > 99%), however, was suppressed. By 8 weeks, while many of the gene changes reverted to normal, the OVX colon still displayed several significant changes: IL‐1*β* expression was reduced, whereas IL‐22 expression was elevated by 13‐fold (CI >95%). Taken together, gene expression analysis of the temporal‐ and region‐specific cytokines suggest that while the small intestine displays only acute (1 and 4 week) cytokine changes to OVX, the colon exhibits long‐term (8 weeks) changes involving IL‐22. Analysis of cytokine gene expression identified that OVX has profound effects on the intestine over surgery, significantly increasing proinflammatory gene expression while suppressing anti‐inflammatory gene expression. The data further revealed that 4 weeks postsurgery is a critical time as this is when the largest OVX effects were observed in all sections of the intestine. Interestingly, the data suggest that 8 weeks postsurgery, the initial effects of estrogen depletion on cytokine expression have returned to levels comparable to the sham mice.

### Correlation of ileal tight junction and cytokine expression

**Figure 8:**
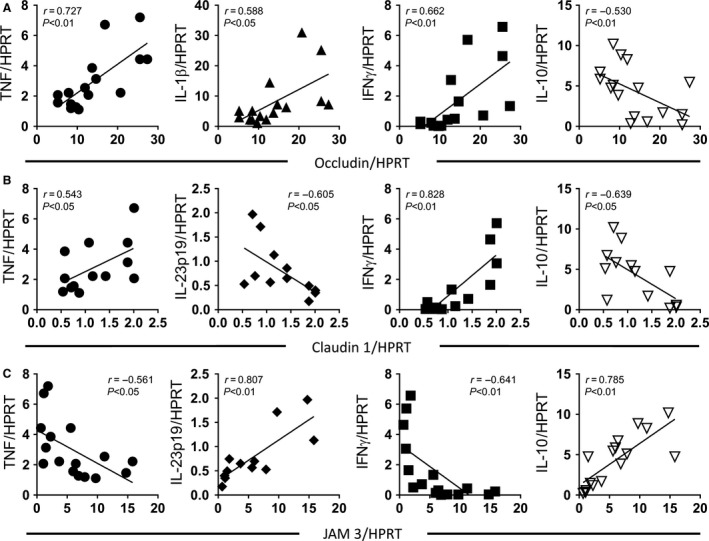
Changes in ileal inflammatory cytokine expression correlate with tight junction gene expression. Female BALB/c mice (12 weeks) underwent either sham or ovariectomy (OVX) surgery. Intestinal regions were isolated and pro‐ and anti‐inflammatory cytokine gene expression analyzed by qPCR. Ileum inflammatory cytokine gene expression (four week postsurgery) was correlated with (A) occludin, (B) claudin 1, and (C) JAM3. (A) TNF *α* (*r* = 0.727, *P *<* *0.01), IL‐1*β* (*r* = 0.588, *P *<* *0.05), and IFN *γ* (*r* = 0.662, *P *<* *0.01) positively correlated with occludin expression, while IL‐10 exhibited a negative correlation (*r* = −0.530, *P *<* *0.01). (B) TNF *α* (*r* = 0.543, *P *<* *0.05) and IFN *γ* (*r* = 0.828, *P *<* *0.01) positively correlated with claudin 1 expression, while a negative correlation was observed with IL‐10 (*r* = −0.639, *P *<* *0.05) and IL‐23p19 (*r* = −0.605, *P *<* *0.05). (C) Analysis of JAM3 expression identified a negative correlation with TNF *α* (*r* = −0.561, *P *<* *0.05) and IFN *γ* (*r* = −0.641, *P *<* *0.01) and a positive correlation with IL‐10 (*r* = 0.785, *P *<* *0.01) and IL‐23p19 (*r* = 0.807, *P *<* *0.01). Statistical analysis performed by Pearson's correlation. *N* = 15–18.

To determine whether the OVX‐induced changes in tight junction gene expression were linked to changes in cytokine gene expression, Pearson's correlation analysis was performed on the 4‐week data. As the ileum exhibited the most dynamic changes in ex vivo permeability, across the time course, we set out to identify a possible link between ileal gene expression and permeability. At the 4‐week time point, elevated ileal occludin expression (Fig. 8A) was significantly and positively correlated with the elevated expression of TNF*α* (*r* = 0.727, *P *<* *0.01), IL‐1*β* (*r* = 0.588, *P *<* *0.05), and IFN*γ* (*r* = 0.662, *P *<* *0.01). In contrast, occludin was negatively correlated with IL‐10 expression (*r* = −0.530, *P *<* *0.01). Compared to occludin, elevated expression of claudin‐1 (Fig. 8B) also correlated with elevated TNF*α* (*r* = 0.56, *P *<* *0.05) and IFN*γ* (*r* = 0.828, *P *<* *0.01), while it correlated negatively with expression of IL‐23p19 (*r* = −0.605, *P *<* *0.05) and IL‐10 (*r* = −0.639, *P *<* *0.05). The observed reduction in JAM3 (Fig 8C) negatively correlated with TNF*α* (*r* = −0.561, *P *<* *0.05) and IFN*γ* (*r* = −0.641, *P *<* *0.01), and positively correlated with IL‐23p19 (*r* = 0.807, *P *<* *0.01) and IL‐10 (*r* = 0.785, *P *<* *0.01).

Analysis of the other intestinal sections identified numerous correlations between cytokine expression and tight junction gene expression (Tables 3, 4, 5, 6, 7, 8, 9, 10). Interestingly, at the 4‐week time point, occludin was found to correlate with expression of TNF*α* and IL‐1*β* in the jejunum and colon. Furthermore, JAM3 was found to correlate with colonic IL‐10 expression, suggesting that these cytokines likely play an important role in the expression of these tight junction proteins.

**Table 3:** Four‐week duodenum tight junction and cytokine correlations: Pearson's *r* values

	TNF*α*	TGF*β*	IL‐1*β*	IFN*γ*	IL‐10	IL‐17A	IL‐22	IL‐23	Occludin	Claudin 1	Claudin 2	Claudin 3	Claudin 4	Claudin 5	Claudin 8	ZO‐1	JAM3
TNF*α*		0.61	0.13	0.53	0.38	−0.14	0.23	−0.10	0.39	−0.07	−0.13	0.30	−0.31	0.75	−0.21	−0.25	−0.07
TGF*β*	0.61		−0.14	0.13	0.55	0.44	0.21	−0.35	0.60	−0.26	0.24	0.09	−0.10	0.49	0.11	−0.02	0.44
IL‐1*β*	0.13	−0.14		−0.02	−0.14	−0.36	0.00	0.10	−0.33	0.20	0.05	0.04	0.08	0.20	−0.30	−0.24	−0.25
IFN*γ*	0.53	0.13	−0.02		0.16	−0.38	−0.03	−0.30	0.13	−0.10	−0.58	0.53	−0.13	0.60	−0.41	−0.46	−0.49
IL‐10	0.38	0.55	−0.14	0.16		−0.06	0.17	−0.24	0.68	−0.39	−0.38	0.09	−0.40	0.43	0.00	0.08	0.50
IL‐17A	−0.14	0.44	−0.36	−0.38	−0.06		0.50	0.04	0.15	0.20	0.61	−0.13	−0.02	−0.14	0.44	0.27	0.46
IL‐22	0.23	0.21	0.00	−0.03	0.17	0.50		−0.47	0.01	0.32	0.28	−0.29	0.02	0.26	0.44	−0.08	0.30
IL‐23	−0.10	−0.35	0.10	−0.30	−0.24	0.04	−0.47		−0.20	0.47	0.05	0.27	−0.12	−0.16	−0.09	0.30	−0.31
Occludin	0.39	0.60	−0.33	0.13	0.68	0.15	0.01	−0.20		−0.54	−0.18	−0.09	−0.37	0.57	−0.01	−0.01	0.37
Claudin 1	−0.07	−0.26	0.20	−0.10	−0.39	0.20	0.32	0.47	−0.54		0.42	0.35	0.38	−0.13	−0.04	0.05	−0.40
Claudin 2	−0.13	0.24	0.05	−0.58	−0.38	0.61	0.28	0.05	−0.18	0.42		−0.27	0.35	−0.30	0.50	0.19	0.03
Claudin 3	0.30	0.09	0.04	0.53	0.09	−0.13	−0.29	0.27	−0.09	0.35	−0.27		−0.07	0.29	−0.21	0.15	−0.44
Claudin 4	−0.31	−0.10	0.08	−0.13	−0.40	−0.02	0.02	−0.12	−0.37	0.38	0.35	−0.07		−0.25	0.20	−0.13	−0.02
Claudin 5	0.75	0.49	0.20	0.60	0.43	−0.14	0.26	−0.16	0.57	−0.13	−0.30	0.29	−0.25		−0.24	−0.36	−0.02
Claudin 8	−0.21	0.11	−0.30	−0.41	0.00	0.44	0.44	−0.09	−0.01	−0.04	0.50	−0.21	0.20	−0.24		0.34	0.24
ZO‐1	−0.25	−0.02	−0.24	−0.46	0.08	0.27	−0.08	0.30	−0.01	0.05	0.19	0.15	−0.13	−0.36	0.34		−0.06
JAM3	−0.07	0.44	−0.25	−0.49	0.50	0.46	0.30	−0.31	0.37	−0.40	0.03	−0.44	−0.02	−0.02	0.24	−0.06	

**Table 4:** Four‐week duodenum tight junction and cytokine correlations: *P* values

	TNF*α*	TGF*β*	IL‐1*β*	IFN*γ*	IL‐10	IL‐17A	IL‐22	IL‐23	Occludin	Claudin 1	Claudin 2	Claudin 3	Claudin 4	Claudin 5	Claudin 8	ZO‐1	JAM3
TNF*α*		0.01	0.61	0.04	0.12	0.60	0.42	0.71	0.12	0.79	0.63	0.24	0.23	0.00	0.43	0.34	0.79
TGF*β*	0.01		0.59	0.63	0.02	0.09	0.45	0.18	0.01	0.31	0.35	0.72	0.71	0.05	0.69	0.93	0.07
IL‐1*β*	0.61	0.59		0.95	0.57	0.17	0.99	0.70	0.19	0.45	0.86	0.89	0.76	0.44	0.26	0.36	0.31
IFN*γ*	0.04	0.63	0.95		0.54	0.18	0.92	0.29	0.64	0.72	0.02	0.04	0.65	0.02	0.15	0.08	0.05
IL‐10	0.12	0.02	0.57	0.54		0.83	0.56	0.37	0.00	0.12	0.13	0.73	0.11	0.08	0.99	0.76	0.04
IL‐17A	0.60	0.09	0.17	0.18	0.83		0.07	0.89	0.61	0.45	0.01	0.62	0.93	0.61	0.10	0.31	0.07
IL‐22	0.42	0.45	0.99	0.92	0.56	0.07		0.10	0.96	0.27	0.32	0.31	0.93	0.37	0.13	0.79	0.28
IL‐23	0.71	0.18	0.70	0.29	0.37	0.89	0.10		0.47	0.07	0.84	0.32	0.65	0.55	0.74	0.26	0.24
Occludin	0.12	0.01	0.19	0.64	0.00	0.61	0.96	0.47		0.03	0.52	0.75	0.16	0.02	0.97	0.98	0.14
Claudin 1	0.79	0.31	0.45	0.72	0.12	0.45	0.27	0.07	0.03		0.10	0.16	0.13	0.62	0.87	0.85	0.11
Claudin 2	0.63	0.35	0.86	0.02	0.13	0.01	0.32	0.84	0.52	0.10		0.29	0.17	0.24	0.05	0.48	0.90
Claudin 3	0.24	0.72	0.89	0.04	0.73	0.62	0.31	0.32	0.75	0.16	0.29		0.80	0.27	0.44	0.56	0.08
Claudin 4	0.23	0.71	0.76	0.65	0.11	0.93	0.93	0.65	0.16	0.13	0.17	0.80		0.34	0.45	0.62	0.94
Claudin 5	0.00	0.05	0.44	0.02	0.08	0.61	0.37	0.55	0.02	0.62	0.24	0.27	0.34		0.37	0.15	0.94
Claudin 8	0.43	0.69	0.26	0.15	0.99	0.10	0.13	0.74	0.97	0.87	0.05	0.44	0.45	0.37		0.20	0.37
ZO‐1	0.34	0.93	0.36	0.08	0.76	0.31	0.79	0.26	0.98	0.85	0.48	0.56	0.62	0.15	0.20		0.83
JAM3	0.79	0.07	0.31	0.05	0.04	0.07	0.28	0.24	0.14	0.11	0.90	0.08	0.94	0.94	0.37	0.83	

**Table 5:** Four‐week jejunum tight junction and cytokine correlations: Pearson's *r* values

	TNF*α*	TGF*β*	IL‐1*β*	IFN*γ*	IL‐10	IL‐17A	IL‐22	IL‐23	Occludin	Claudin 1	Claudin 2	Claudin 3	Claudin 4	Claudin 5	Claudin 8	ZO‐1	JAM3
TNF*α*		0.17	0.54	0.63	−0.11	0.23	0.21	−0.51	0.51	0.05	0.61	−0.24	−0.20	−0.59	−0.40	−0.30	−0.28
TGF*β*	0.17		0.42	0.27	0.43	−0.31	0.73	−0.39	0.74	0.27	0.35	−0.55	0.28	−0.32	−0.37	0.06	0.63
IL‐1*β*	0.54	0.42		0.72	−0.35	0.03	0.40	−0.48	0.72	0.02	0.17	0.12	0.16	−0.42	−0.66	−0.19	−0.23
IFN*γ*	0.63	0.27	0.72		−0.33	0.12	0.03	−0.40	0.41	0.53	0.34	−0.23	0.25	−0.41	−0.54	−0.52	−0.42
IL‐10	−0.11	0.43	−0.35	−0.33		−0.28	0.23	0.08	0.18	−0.28	−0.07	−0.22	0.02	−0.13	0.24	0.44	0.73
IL‐17A	0.23	−0.31	0.03	0.12	−0.28		−0.62	−0.06	−0.22	0.00	0.20	0.26	−0.39	−0.28	0.00	0.08	−0.44
IL‐22	0.21	0.73	0.40	0.03	0.23	−0.62		−0.17	0.57	−0.23	0.75	−0.21	0.21	−0.29	−0.26	0.03	0.43
IL‐23	−0.51	−0.39	−0.48	−0.40	0.08	−0.06	−0.17		−0.60	0.02	0.10	0.18	−0.06	0.35	0.25	0.41	−0.19
Occludin	0.51	0.74	0.72	0.41	0.18	−0.22	0.57	−0.60		−0.06	0.50	−0.19	0.32	−0.36	−0.55	0.00	0.40
Claudin 1	0.05	0.27	0.02	0.53	−0.28	0.00	−0.23	0.02	−0.06		0.08	−0.61	−0.01	0.03	0.08	−0.38	−0.21
Claudin 2	0.61	0.35	0.17	0.34	−0.07	0.20	0.75	0.10	0.50	0.08		−0.22	0.09	−0.04	−0.32	−0.14	0.12
Claudin 3	−0.24	−0.55	0.12	−0.23	−0.22	0.26	−0.21	0.18	−0.19	−0.61	−0.22		0.30	0.36	−0.32	0.13	−0.26
Claudin 4	−0.20	0.28	0.16	0.25	0.02	−0.39	0.21	−0.06	0.32	−0.01	0.09	0.30		0.52	−0.48	−0.18	0.23
Claudin 5	−0.59	−0.32	−0.42	−0.41	−0.13	−0.28	−0.29	0.35	−0.36	0.03	−0.04	0.36	0.52		0.11	−0.06	0.07
Claudin 8	−0.40	−0.37	−0.66	−0.54	0.24	0.00	−0.26	0.25	−0.55	0.08	−0.32	−0.32	−0.48	0.11		0.33	0.04
ZO‐1	−0.30	0.06	−0.19	−0.52	0.44	0.08	0.03	0.41	0.00	−0.38	−0.14	0.13	−0.18	−0.06	0.33		0.45
JAM3	−0.28	0.63	−0.23	−0.42	0.73	−0.44	0.43	−0.19	0.40	−0.21	0.12	−0.26	0.23	0.07	0.04	0.45	

**Table 6:** Four‐week jejunum tight junction and cytokine correlations: *P* values

	TNF*α*	TGF*β*	IL‐1*β*	IFN*γ*	IL‐10	IL‐17A	IL‐22	IL‐23	Occludin	Claudin 1	Claudin 2	Claudin 3	Claudin 4	Claudin 5	Claudin 8	ZO‐1	JAM3
TNF*α*		0.52	0.05	0.01	0.68	0.50	0.48	0.07	0.04	0.87	0.02	0.43	0.50	0.03	0.16	0.30	0.30
TGF*β*	0.52		0.14	0.32	0.10	0.35	0.00	0.17	0.00	0.35	0.22	0.05	0.33	0.27	0.20	0.83	0.01
IL‐1*β*	0.05	0.14		0.00	0.22	0.94	0.20	0.12	0.00	0.95	0.60	0.73	0.63	0.18	0.02	0.56	0.43
IFN*γ*	0.01	0.32	0.00		0.21	0.73	0.92	0.16	0.11	0.05	0.24	0.44	0.38	0.15	0.05	0.05	0.11
IL‐10	0.68	0.10	0.22	0.21		0.41	0.42	0.78	0.51	0.33	0.82	0.48	0.94	0.65	0.40	0.12	0.00
IL‐17A	0.50	0.35	0.94	0.73	0.41		0.05	0.84	0.51	0.99	0.54	0.44	0.21	0.38	1.00	0.81	0.18
IL‐22	0.48	0.00	0.20	0.92	0.42	0.05		0.60	0.03	0.48	0.00	0.53	0.50	0.36	0.41	0.94	0.12
IL‐23	0.07	0.17	0.12	0.16	0.78	0.84	0.60		0.02	0.95	0.74	0.54	0.83	0.20	0.37	0.13	0.52
Occludin	0.04	0.00	0.00	0.11	0.51	0.51	0.03	0.02		0.85	0.07	0.52	0.26	0.21	0.04	0.99	0.13
Claudin 1	0.87	0.35	0.95	0.05	0.33	0.99	0.48	0.95	0.85		0.78	0.02	0.97	0.92	0.78	0.16	0.46
Claudin 2	0.02	0.22	0.60	0.24	0.82	0.54	0.00	0.74	0.07	0.78		0.46	0.76	0.90	0.25	0.62	0.67
Claudin 3	0.43	0.05	0.73	0.44	0.48	0.44	0.53	0.54	0.52	0.02	0.46		0.30	0.21	0.26	0.65	0.39
Claudin 4	0.50	0.33	0.63	0.38	0.94	0.21	0.50	0.83	0.26	0.97	0.76	0.30		0.05	0.07	0.52	0.43
Claudin 5	0.03	0.27	0.18	0.15	0.65	0.38	0.36	0.20	0.21	0.92	0.90	0.21	0.05		0.70	0.84	0.80
Claudin 8	0.16	0.20	0.02	0.05	0.40	1.00	0.41	0.37	0.04	0.78	0.25	0.26	0.07	0.70		0.23	0.90
ZO‐1	0.30	0.83	0.56	0.05	0.12	0.81	0.94	0.13	0.99	0.16	0.62	0.65	0.52	0.84	0.23		0.11
JAM3	0.30	0.01	0.43	0.11	0.00	0.18	0.12	0.52	0.13	0.46	0.67	0.39	0.43	0.80	0.90	0.11	

**Table 7:** Four‐week ileum tight junction and cytokine correlations: Pearson's *r* values

	TNF*α*	TGF*β*	IL‐1*β*	IFN*γ*	IL‐10	IL‐17A	IL‐22	IL‐23	Occludin	Claudin 1	Claudin 2	Claudin 3	Claudin 4	Claudin 5	Claudin 8	ZO‐1	JAM3
TNF*α*		−0.38	0.18	0.87	−0.57	0.34	0.18	−0.46	0.73	0.54	−0.19	0.32	−0.05	0.22	0.62	−0.10	−0.56
TGF*β*	−0.38		0.09	−0.58	0.61	−0.04	0.42	0.41	−0.04	−0.40	0.17	0.25	0.23	−0.04	−0.44	−0.16	0.32
IL‐1*β*	0.18	0.09		0.35	−0.54	−0.28	−0.10	−0.41	0.59	0.53	0.09	−0.12	0.07	−0.16	−0.29	−0.14	−0.49
IFN*γ*	0.87	−0.58	0.35		−0.67	0.15	−0.06	−0.52	0.66	0.83	−0.22	−0.01	−0.07	0.15	0.56	−0.11	−0.64
IL‐10	−0.57	0.61	−0.54	−0.67		−0.06	0.14	0.84	−0.53	−0.64	−0.01	−0.01	0.29	0.21	−0.25	−0.07	0.79
IL‐17A	0.34	−0.04	−0.28	0.15	−0.06		0.70	−0.21	0.05	0.21	0.62	0.75	−0.27	−0.11	0.52	−0.03	0.14
IL‐22	0.18	0.42	−0.10	−0.06	0.14	0.70		−0.10	0.30	0.06	0.42	0.77	−0.28	−0.40	0.14	−0.37	0.13
IL‐23	−0.46	0.41	−0.41	−0.52	0.84	−0.21	−0.10		−0.47	−0.60	−0.07	−0.04	0.64	0.39	−0.12	0.07	0.81
Occludin	0.73	−0.04	0.59	0.66	−0.53	0.05	0.30	−0.47		0.53	−0.23	0.32	0.00	−0.06	0.16	−0.41	−0.55
Claudin 1	0.54	−0.40	0.53	0.83	−0.64	0.21	0.06	−0.60	0.53		0.19	0.14	−0.08	−0.21	0.28	0.00	−0.57
Claudin 2	−0.19	0.17	0.09	−0.22	−0.01	0.62	0.42	−0.07	−0.23	0.19		0.31	−0.06	−0.26	0.30	0.22	0.27
Claudin 3	0.32	0.25	−0.12	−0.01	−0.01	0.75	0.77	−0.04	0.32	0.14	0.31		−0.02	−0.15	0.14	−0.08	0.14
Claudin 4	−0.05	0.23	0.07	−0.07	0.29	−0.27	−0.28	0.64	0.00	−0.08	−0.06	−0.02		0.43	−0.07	0.10	0.41
Claudin 5	0.22	−0.04	−0.16	0.15	0.21	−0.11	−0.40	0.39	−0.06	−0.21	−0.26	−0.15	0.43		0.30	0.20	0.14
Claudin 8	0.62	−0.44	−0.29	0.56	−0.25	0.52	0.14	−0.12	0.16	0.28	0.30	0.14	−0.07	0.30		0.23	−0.14
ZO‐1	−0.10	−0.16	−0.14	−0.11	−0.07	−0.03	−0.37	0.07	−0.41	0.00	0.22	−0.08	0.10	0.20	0.23		0.00
JAM3	−0.56	0.32	−0.49	−0.64	0.79	0.14	0.13	0.81	−0.55	−0.57	0.27	0.14	0.41	0.14	−0.14	0.00	

**Table 8:** Four‐week ileum tight junction and cytokine correlations: *P* values

	TNF*α*	TGF*β*	IL‐1*β*	IFN*γ*	IL‐10	IL‐17A	IL‐22	IL‐23	Occludin	Claudin 1	Claudin 2	Claudin 3	Claudin 4	Claudin 5	Claudin 8	ZO‐1	JAM3
TNF*α*		0.14	0.51	0.00	0.02	0.24	0.51	0.14	0.00	0.06	0.53	0.26	0.86	0.46	0.02	0.73	0.02
TGF*β*	0.14		0.74	0.02	0.01	0.90	0.11	0.18	0.90	0.17	0.55	0.39	0.42	0.90	0.11	0.58	0.22
IL‐1*β*	0.51	0.74		0.19	0.03	0.33	0.71	0.19	0.02	0.06	0.77	0.67	0.82	0.57	0.32	0.64	0.06
IFN*γ*	0.00	0.02	0.19		0.00	0.61	0.82	0.09	0.01	0.00	0.45	0.99	0.82	0.62	0.04	0.71	0.01
IL‐10	0.02	0.01	0.03	0.00		0.85	0.60	0.00	0.03	0.02	0.98	0.97	0.31	0.48	0.39	0.80	0.00
IL‐17A	0.24	0.90	0.33	0.61	0.85		0.01	0.49	0.86	0.47	0.01	0.00	0.33	0.69	0.05	0.90	0.64
IL‐22	0.51	0.11	0.71	0.82	0.60	0.01		0.75	0.26	0.83	0.13	0.00	0.33	0.15	0.63	0.19	0.62
IL‐23	0.14	0.18	0.19	0.09	0.00	0.49	0.75		0.12	0.04	0.82	0.90	0.02	0.19	0.69	0.81	0.00
Occludin	0.00	0.90	0.02	0.01	0.03	0.86	0.26	0.12		0.06	0.43	0.27	1.00	0.85	0.59	0.15	0.03
Claudin 1	0.06	0.17	0.06	0.00	0.02	0.47	0.83	0.04	0.06		0.51	0.62	0.78	0.47	0.33	0.99	0.04
Claudin 2	0.53	0.55	0.77	0.45	0.98	0.01	0.13	0.82	0.43	0.51		0.26	0.84	0.35	0.28	0.44	0.35
Claudin 3	0.26	0.39	0.67	0.99	0.97	0.00	0.00	0.90	0.27	0.62	0.26		0.95	0.59	0.63	0.78	0.62
Claudin 4	0.86	0.42	0.82	0.82	0.31	0.33	0.33	0.02	1.00	0.78	0.84	0.95		0.11	0.80	0.72	0.15
Claudin 5	0.46	0.90	0.57	0.62	0.48	0.69	0.15	0.19	0.85	0.47	0.35	0.59	0.11		0.28	0.47	0.64
Claudin 8	0.02	0.11	0.32	0.04	0.39	0.05	0.63	0.69	0.59	0.33	0.28	0.63	0.80	0.28		0.40	0.63
ZO‐1	0.73	0.58	0.64	0.71	0.80	0.90	0.19	0.81	0.15	0.99	0.44	0.78	0.72	0.47	0.40		1.00
JAM3	0.02	0.22	0.06	0.01	0.00	0.64	0.62	0.00	0.03	0.04	0.35	0.62	0.15	0.64	0.63	1.00	

**Table 9:** Four‐week colon tight junction and cytokine correlations: Pearson's *r* values

	TNF*α*	TGF*β*	IL‐1*β*	IFN*γ*	IL‐10	IL‐17A	IL‐22	IL‐23	Occludin	Claudin 1	Claudin 2	Claudin 3	Claudin 4	Claudin 5	Claudin 8	ZO‐1	JAM3
TNF*α*		0.28	0.33	0.84	−0.33	0.03	0.66	−0.33	0.70	0.03	0.13	0.12	0.26	−0.01	−0.04	−0.53	−0.21
TGF*β*	0.28		0.22	0.47	0.12	0.23	−0.46	−0.03	0.52	0.05	−0.10	0.06	−0.39	0.40	0.06	−0.09	0.20
IL‐1*β*	0.33	0.22		0.42	−0.62	0.11	0.04	−0.30	0.64	−0.23	−0.13	0.02	−0.40	0.17	−0.09	−0.33	−0.50
IFN*γ*	0.84	0.47	0.42		−0.51	0.16	0.17	−0.19	0.79	0.18	−0.03	−0.05	−0.11	0.03	−0.12	−0.56	−0.26
IL‐10	−0.33	0.12	−0.62	−0.51		−0.12	−0.45	0.33	−0.36	0.31	0.11	0.50	−0.02	0.00	0.47	0.43	0.93
IL‐17A	0.03	0.23	0.11	0.16	−0.12		−0.14	−0.11	−0.08	0.02	0.00	0.09	−0.10	−0.30	0.20	0.37	−0.30
IL‐22	0.66	−0.46	0.04	0.17	−0.45	−0.14		−0.23	0.07	−0.04	0.11	0.01	0.52	−0.45	−0.21	−0.19	−0.48
IL‐23	−0.33	−0.03	−0.30	−0.19	0.33	−0.11	−0.23		−0.37	0.78	0.69	0.07	0.30	0.09	0.30	0.76	0.36
Occludin	0.70	0.52	0.64	0.79	−0.36	−0.08	0.07	−0.37		−0.08	−0.23	0.05	−0.14	0.39	−0.23	−0.66	−0.19
Claudin 1	0.03	0.05	−0.23	0.18	0.31	0.02	−0.04	0.78	−0.08		0.72	0.27	0.40	0.10	0.22	0.47	0.20
Claudin 2	0.13	−0.10	−0.13	−0.03	0.11	0.00	0.11	0.69	−0.23	0.72		−0.10	0.57	−0.01	0.34	0.42	0.10
Claudin 3	0.12	0.06	0.02	−0.05	0.50	0.09	0.01	0.07	0.05	0.27	−0.10		−0.09	0.07	0.63	0.30	0.40
Claudin 4	0.26	−0.39	−0.40	−0.11	−0.02	−0.10	0.52	0.30	−0.14	0.40	0.57	−0.09		−0.03	−0.21	0.13	−0.09
Claudin 5	−0.01	0.40	0.17	0.03	0.00	−0.30	−0.45	0.09	0.39	0.10	−0.01	0.07	−0.03		−0.06	−0.15	0.10
Claudin 8	−0.04	0.06	−0.09	−0.12	0.47	0.20	−0.21	0.30	−0.23	0.22	0.34	0.63	−0.21	−0.06		0.44	0.44
ZO‐1	−0.53	−0.09	−0.33	−0.56	0.43	0.37	−0.19	0.76	−0.66	0.47	0.42	0.30	0.13	−0.15	0.44		0.25
JAM3	−0.21	0.20	−0.50	−0.26	0.93	−0.30	−0.48	0.36	−0.19	0.20	0.10	0.40	−0.09	0.10	0.44	0.25	

**Table 10:** Four‐week colon tight junction and cytokine correlations: *P* values

	TNF*α*	TGF*β*	IL‐1*β*	IFN*γ*	IL‐10	IL‐17A	IL‐22	IL‐23	Occludin	Claudin 1	Claudin 2	Claudin 3	Claudin 4	Claudin 5	Claudin 8	ZO‐1	JAM3
TNF*α*		0.27	0.19	0.00	0.22	0.90	0.01	0.22	0.00	0.92	0.65	0.66	0.32	0.96	0.86	0.04	0.41
TGF*β*	0.27		0.38	0.05	0.65	0.41	0.09	0.92	0.03	0.86	0.72	0.84	0.12	0.11	0.81	0.73	0.43
IL‐1*β*	0.19	0.38		0.09	0.01	0.70	0.90	0.25	0.00	0.37	0.65	0.94	0.11	0.51	0.74	0.21	0.04
IFN*γ*	0.00	0.05	0.09		0.05	0.56	0.57	0.49	0.00	0.51	0.91	0.85	0.69	0.93	0.67	0.03	0.33
IL‐10	0.22	0.65	0.01	0.05		0.69	0.11	0.23	0.17	0.24	0.70	0.06	0.93	1.00	0.07	0.11	0.00
IL‐17A	0.90	0.41	0.70	0.56	0.69		0.66	0.70	0.78	0.94	0.99	0.75	0.71	0.27	0.47	0.19	0.28
IL‐22	0.01	0.09	0.90	0.57	0.11	0.66		0.44	0.81	0.89	0.73	0.97	0.06	0.11	0.47	0.53	0.08
IL‐23	0.22	0.92	0.25	0.49	0.23	0.70	0.44		0.15	0.00	0.01	0.80	0.25	0.73	0.26	0.00	0.17
Occludin	0.00	0.03	0.00	0.00	0.17	0.78	0.81	0.15		0.75	0.40	0.86	0.58	0.12	0.39	0.01	0.47
Claudin 1	0.92	0.86	0.37	0.51	0.24	0.94	0.89	0.00	0.75		0.00	0.31	0.11	0.70	0.40	0.06	0.43
Claudin 2	0.65	0.72	0.65	0.91	0.70	0.99	0.73	0.01	0.40	0.00		0.74	0.03	0.96	0.22	0.14	0.73
Claudin 3	0.66	0.84	0.94	0.85	0.06	0.75	0.97	0.80	0.86	0.31	0.74		0.74	0.81	0.01	0.27	0.13
Claudin 4	0.32	0.12	0.11	0.69	0.93	0.71	0.06	0.25	0.58	0.11	0.03	0.74		0.91	0.43	0.63	0.73
Claudin 5	0.96	0.11	0.51	0.93	1.00	0.27	0.11	0.73	0.12	0.70	0.96	0.81	0.91		0.82	0.58	0.70
Claudin 8	0.86	0.81	0.74	0.67	0.07	0.47	0.47	0.26	0.39	0.40	0.22	0.01	0.43	0.82		0.09	0.08
ZO‐1	0.04	0.73	0.21	0.03	0.11	0.19	0.53	0.00	0.01	0.06	0.14	0.27	0.63	0.58	0.09		0.35
JAM3	0.41	0.43	0.04	0.33	0.00	0.28	0.08	0.17	0.47	0.43	0.73	0.13	0.73	0.70	0.08	0.35	

## Discussion

Loss of estrogen leads to many adverse consequences on both reproductive and nonreproductive tissues, including urogenital atrophy and osteoporosis (Burger et al. 1999; Khosla et al. 2011; Davis et al. 2012; Lisabeth and Bushnell 2012; Portman and Gass 2014; Weber et al. 2014). Historically, the main effect associated with estrogen deficiency on the intestine has been calcium malabsorption (Heaney et al. 1989). Recent studies, however, have suggested that loss of estrogen has a more complex effect on intestinal permeability and proinflammatory cytokine expression (Braniste et al. 2009; Li et al. 2016). However, studies to date have not examined the intestinal changes that occur at the onset of estrogen deficiency. In addition, there are no studies that have determined whether these changes are localized to specific intestinal segments and when these changes begin and how long they persist. In the present study, we demonstrate time‐dependent changes in in vivo intestinal permeability following loss of estrogen. We further reveal, ex vivo, that estrogen deficiency results in temporal‐ and regional‐specific changes in permeability, tight junction gene expression, and pro‐ and anti‐inflammatory cytokine gene expression.

The intestinal epithelium provides a critical physiological barrier between the luminal gut microbiome and the host (Turner 2009). Dysregulation of this barrier allows the permeation of pathogens, toxins, and antigens across the mucosal tissue into the systemic sites which can lead to adverse consequences, as seen in inflammatory bowel disease (IBD), celiac disease, and type I diabetes (Arrieta et al. 2006; Turner 2009; Lee 2015). Estrogen receptor beta (ER*β*) is the predominant ER type in the intestinal tract, though expression of ER*α* and the G protein‐coupled estrogen receptor (GPER) have also been reported (Choijookhuu et al. 2016). While the direct effect of OVX on intestinal ER expression is not clear, changes in estrogen levels as observed during the menstrual cycle and pregnancy have been observed to modulate ER levels, with elevated estrogen levels corresponding to increased ER*β* and ER*α* expression (Choijookhuu et al. 2016). Previous studies on the effects of estrogen deficiency on intestinal permeability have not been consistent. Using mice, Li et al. (2016) showed that in vivo intestinal permeability is increased 4 weeks after OVX surgery. However, in a rat model of OVX, no difference in in vivo permeability was observed after 5 weeks (Cox‐York et al. 2015). Our results demonstrate that the onset of enhanced intestinal permeability occurs early after OVX (1 week), but normalizes over time (by 4 and 8 weeks), suggesting that inconsistencies in previous studies may be attributed to studying a single time‐point measurement following surgery. The precise difference in time points of enhanced intestinal permeability between ours and Li et al. could be due to different mouse strains (ours was BALB/c vs. C57BL/6J used by Li et al.). Although such strain‐specific difference in OVX‐induced intestinal permeability has not been reported, differences in BALB/c and C57BL/6 bone density in responses to OVX (Beamer et al. 1996; Bouxsein et al. 2005), susceptibility to organ fibrosis (Walkin et al. 2013), and immune response (Watanabe et al. 2004) have been demonstrated. The current study thus builds upon previous findings by showing that ablation of estrogen modulates in vivo intestinal permeability in a time‐dependent manner, and occurs early after induction of estrogen deficiency.

We demonstrate here for the first time that estrogen deficiency induces region‐specific effects on intestinal permeability. Furthermore, analyses of regional permeability ex vivo correlated with the overall in vivo permeability. Interestingly, while the permeability status of the duodenum (increased) and colon (decreased) was maintained across the time course, the effects on the jejunum and ileum were more dynamic. The time frame of changes in OVX ileal ex vivo permeability, from increased to decreased (compared to sham), matches that of the in vivo changes. This suggests that, in this model, ileum barrier function may have a critical contribution to overall intestinal permeability. The decreased colonic permeability observed in the current study is contrary to those previously reported (Braniste et al. 2009). However, care should be taken before making direct comparisons. Braniste et al. (2009) used Wistar rats that were ovariectomized for an 11‐day period before distal colonic permeability measurements were taken. While the difference could be related to species‐specific response to estrogen deficiency, other possibilities exist as well. In the present study, a distal section of the colon was analyzed for permeability. Differences in proximal and distal colon permeability have been observed both by our laboratory (data not shown) and by other groups in rodents (Fihn and Jodal 2001; Busche et al. 2002). This raises the possibility that dynamic changes in permeability occur within as well as between the sections of the intestine.

To decipher the mechanistic basis of changes in intestinal permeability following estrogen deficiency, we investigated expression of the tight junctions. These are multiple protein complexes located at the apical end of the epithelial cells and determine the paracellular permeability to solutes (Suzuki 2013). Four key transmembrane proteins have been identified: occludin, claudins, JAMs, and tricellulin (Ulluwishewa et al. 2011; Suzuki 2013). Intracellular proteins like the zonula occludins (ZO) are crucial to the assembly of the tight junction as they have multiple sites that interact with occludin and members of the claudin family (Fanning et al. 1998). These transmembrane proteins play important roles in maintaining barrier integrity. Occludin regulates paracellular diffusion, claudin‐1, ‐3, ‐4, ‐5, and ‐8 decrease permeability, while claudin‐2 increases permeability; JAMs modulate permeability, with JAM3 potentially increasing intestinal permeability (Orlova et al. 2006; Li et al. 2009), while tricellulin seals the space between three adjacent cells (Turner 2009; Ulluwishewa et al. 2011; Suzuki 2013). Analysis of gene expression of tight junctions in the current study explains some of the changes observed in specific segmental permeability. For example, the significantly increased duodenal permeability observed at 4 weeks corresponded to a significant decrease in claudin‐8 expression. The increased jejunal permeability at 4 weeks corresponded to decreased claudin‐8 expression. The change from higher ileal permeability to lower permeability over the time course corresponded with increases in occludin, claudin‐1, ‐3, and ‐5. While the reduced OVX colon permeability corresponded with reduced JAM3 expression and increased tight junction gene expression. Previous studies have shown that depletion of estrogen modulates some intestinal tight junction expression (Braniste et al. 2009; Li et al. 2016). In the study by Li et al. (2016), 4 weeks after OVX, C57BL/6 mouse gene expression of claudin‐2, ‐3, ‐15, and JAM3 were significantly reduced in the small intestine. In a separate study, treatment of ovariectomized rats with estradiol significantly increased occludin expression, but had no effect on ZO‐1 (Braniste et al. 2009). Even though some specific observations are different between our studies (possibly due to strain and time points), some similarities in results exist; at 4 weeks, JAM3 expression was reduced in the ileum, while at 1 week, occludin expression was reduced in the colon, whereas ZO‐1 exhibited no significant differences. Taken together, these data highlight that estrogen and its subsequent loss has a major impact on barrier integrity and this is exquisitely dependent on time after induction of deficiency and the specific region of the intestine.

To further establish the mechanism behind the estrogen deficiency‐induced changes in tight junction gene expression, we examined the effect of OVX on intestinal cytokine gene expression. Estrogen loss is associated with increased proinflammatory cytokine expression, both systemically and in the bone marrow (Bismar et al. 1995; Malutan et al. 2014; Collins et al. 2016). The effects of OVX on intestinal cytokine expression were found to be complex with both regional and temporal changes in pro‐ and anti‐inflammatory cytokines. Consistent with previous reports, OVX increased TNF*α* expression 4 weeks postsurgery. However, in contrast to previous reports (Li et al. 2016), no change in IL‐17A expression was identified at the 4 week time point, though levels were elevated in the OVX mice 1 week postsurgery, suggesting possible immune differences in the mouse strains used (Watanabe et al. 2004). Our results also suggest that the changes in inflammatory cytokine expression are likely responsible for the modulation of the tight junction expression. In the ileum and colon, increased gene expression of TNF*α* and IL‐1*β* were found to correlate with increased expression of occludin. In addition, expression of IL‐10 was found to correlate with that of JAM3, with both being downregulated in the OVX condition. This suggests that specific cytokines likely modulate specific tight junctions to help maintain barrier integrity. The positive correlation between TNF*α*, IL‐1*β*, occludin, and decreased permeability appears contrary to previously published in vitro data that showed high concentrations of TNF*α* and IL‐1*β* increases Caco‐2 cell permeability (Ma et al. 2005; Al‐Sadi et al. 2008). However, these studies were modeling IBD, which is characterized by strikingly high expression of these proinflammatory cytokines (Ma et al. 2005; Al‐Sadi et al. 2008; Strober and Fuss 2011). Changes in proinflammatory cytokine expression following loss of estrogen are modest, even though statistically significant. Rather than being destructive, a modest increase in intestinal cytokine expression could be a protective mechanism. Inflammatory cytokines, such as TNF*α* and IL‐1*β*, are known to induce beneficial as well as detrimental effects on numerous cell types by modulating tight junctions in tubular epithelial cells (Amoozadeh et al. 2015), regulating human neutrophil apoptosis (van den Berg et al. 2001), and stimulating osteoblast mineralization or inhibiting osteoblast proliferation (Lin et al. 2010). Thus, it is possible that the increased cytokine gene expression and subsequent increase in tight junction gene expression is to help maintain barrier integrity, and therefore, prevent translocation of harmful bacteria. However, it is also possible that some of the changes in cytokine gene expression and that of tight junction genes are independent and stimulated by microbial factors in the different segments subsequent to estrogen deficiency.

Interestingly, the temporal change in intestinal inflammatory cytokine gene expression, increasing over 1 and 4 weeks before returning to normal levels at 8 weeks, is analogous to observed cytokine expression in menopausal women. Studies have shown that expression of IL‐1*β* in monocyte cultures increase in the first years after menopause before returning to premenopausal levels (Pacifici et al. 1993). Furthermore, bone marrow cultures isolated from early postmenopausal women expressed increased levels of IL‐1*α*, TNF*α*, and IL‐6 compared to premenopausal women, whereas no difference was observed between pre‐ and late postmenopausal women (Bismar et al. 1995; Pfeilschifter et al. 2002).

In summary, the results from this study demonstrate for the first time that loss of estrogen has regional and temporal effects on the intestine. We show, in vivo, that intestinal permeability increases early after OVX surgery before returning to sham levels, and ex vivo, the permeability of the different intestinal regions differs. Furthermore, we reveal temporal and regional changes in tight junction gene expression correlating with inflammatory genes and subsequent changes in permeability. These results demonstrate that estrogen deficiency induces a new and altered state in the gastrointestinal system that eventually normalizes for some physiological observations, likely due to complex compensatory mechanisms. Given that estrogen affects almost all physiological processes, our studies indicate that the effects of estrogen deficiency are time dependent in the intestine and single time‐point measurements of intestinal function could be misleading.

## Conflict of Interest

None declared.

## References

[phy213263-bib-0001] Al‐Sadi, R. , D. Ye , K. Dokladny , and T. Y. Ma . 2008 Mechanism of IL‐1beta‐induced increase in intestinal epithelial tight junction permeability. J. Immunol. 180:5653–5661. doi: 180/8/5653 [pii]. 18390750 10.4049/jimmunol.180.8.5653 PMC3035485

[phy213263-bib-0002] Amoozadeh, Y. , Q. Dan , J. Xiao , F. Waheed , and K. Szászi . 2015 Tumor necrosis factor‐*α* induces a biphasic change in claudin‐2 expression in tubular epithelial cells: role in barrier functions. Am. J. Physiol. Cell Physiol. 309:C38–C50. 25948735 10.1152/ajpcell.00388.2014 PMC4490324

[phy213263-bib-0003] Arrieta, M. C. , L. Bistritz , and J. B. Meddings . 2006 Alterations in intestinal permeability. Gut 55:1512–1520. 16966705 10.1136/gut.2005.085373 PMC1856434

[phy213263-bib-0004] Babaei, P. , R. Mehdizadeh , M. M. Ansar , and A. Damirchi . 2010 Effects of ovariectomy and estrogen replacement therapy on visceral adipose tissue and serum adiponectin levels in rats. Menopause Int. 16:100–104. 20956683 10.1258/mi.2010.010028

[phy213263-bib-0005] Beamer, W. G. , L. R. Donahue , C. J. Rosen , and D. J. Baylink . 1996 Genetic variability in adult bone density among inbred strains of mice. Bone 18:397–403. 8739896 10.1016/8756-3282(96)00047-6

[phy213263-bib-0006] van den Berg, J. M. , S. Weyer , J. J. Weening , D. Roos , and T. W. Kuijpers . 2001 Divergent effects of tumor necrosis factor alpha on apoptosis of human neutrophils. J. Leukoc. Biol. 69:467–473. 11261795

[phy213263-bib-0007] Bismar, H. , I. Diel , R. Ziegler , and J. Pfeilschifter . 1995 Increased cytokine secretion by human bone marrow cells after menopause or discontinuation of estrogen replacement. J. Clin. Endocrinol. Metab. 80:3351–3355. 7593450 10.1210/jcem.80.11.7593450

[phy213263-bib-0008] Bouxsein, M. L. , K. S. Myers , K. L. Shultz , L. R. Donahue , C. J. Rosen , and W. G. Beamer . 2005 Ovariectomy‐induced bone loss varies among inbred strains of mice. J. Bone and Miner. Res. 20:1085–1092. 15940361 10.1359/JBMR.050307

[phy213263-bib-0009] Braniste, V. , M. Leveque , C. Buisson‐Brenac , L. Bueno , J. Fioramonti , and E. Houdeau . 2009 Oestradiol decreases colonic permeability through oestrogen receptor beta‐mediated up‐regulation of occludin and junctional adhesion molecule‐A in epithelial cells. J. Physiol. 587(Pt 13):3317–3328. 19433574 10.1113/jphysiol.2009.169300 PMC2727039

[phy213263-bib-0010] Burger, H. G. , E. C. Dudley , J. L. Hopper , N. Groome , J. R. Guthrie , A. Green , et al. 1999 Prospectively measured levels of serum follicle‐stimulating hormone, estradiol, and the dimeric inhibins during the menopausal transition in a population‐based cohort of women. J. Clin. Endocrinol. Metab. 84:4025–4030. 10566644 10.1210/jcem.84.11.6158

[phy213263-bib-0011] Busche, R. , J. Dittmann , H. D. Meyer zu Düttingdorf , U. Glockenthör , W. Von Engelhardt , and H. P. Sallmann . 2002 Permeability properties of apical and basolateral membranes of the guinea pig caecal and colonic epithelia for short‐chain fatty acids. Biochim. Biophys. Acta 1565:55–63. 12225852 10.1016/s0005-2736(02)00505-9

[phy213263-bib-0012] Choijookhuu, N. , S. Hino , P. S. Oo , B. Batmunkh , and Y. Hishikawa . 2016 The role of estrogen receptors in intestinal homeostasis and disease. Receptor Clin. Investig. 3:1–8.

[phy213263-bib-0013] Collins, F. L. , R. Irwin , H. Bierhalter , J. Schepper , R. A. Britton , N. Parameswaran , et al. 2016 Lactobacillus reuteri 6475 Increases Bone Density in Intact Females Only under an Inflammatory Setting. PLoS ONE 11:e0153180. 27058036 10.1371/journal.pone.0153180 PMC4825993

[phy213263-bib-0014] Cox‐York, K. A. , A. M. Sheflin , M. T. Foster , C. L. Gentile , A. Kahl , L. G. Koch , et al. 2015 Ovariectomy results in differential shifts in gut microbiota in low versus high aerobic capacity rats. Physiol. Rep. 3. doi: 10.14814/phy2.12488 10.14814/phy2.12488 PMC4562574 26265751

[phy213263-bib-0015] Davis, S. R. , C. Castelo‐Branco , P. Chedraui , M. A. Lumsden , R. E. Nappi , D. Shah , et al. 2012 Understanding weight gain at menopause. Climacteric 15:419–429. 22978257 10.3109/13697137.2012.707385

[phy213263-bib-0016] Davis, S. R. , I. Lambrinoudaki , M. Lumsden , G. D. Mishra , L. Pal , M. Rees , et al. 2015 Menopause. Nat. Rev. Dis. Primers 1:15004. 27188659 10.1038/nrdp.2015.4

[phy213263-bib-0017] Fanning, A. S. , B. J. Jameson , L. A. Jesaitis , and J. M. Anderson . 1998 The tight junction protein ZO‐1 establishes a link between the transmembrane protein occludin and the actin cytoskeleton. J. Biol. Chem. 273:29745–29753. 9792688 10.1074/jbc.273.45.29745

[phy213263-bib-0018] Fihn, B. M. , and M. Jodal . 2001 Permeability of the proximal and distal rat colon crypt and surface epithelium to hydrophilic molecules. Pflugers Arch. 441:656–662. 11294247 10.1007/s004240000440

[phy213263-bib-0019] Heaney, R. P. , R. R. Recker , M. R. Stegman , and A. J. Moy . 1989 Calcium absorption in women: relationships to calcium intake, estrogen status, and age. J. Bone and Miner. Res. 4:469–475. 2816496 10.1002/jbmr.5650040404

[phy213263-bib-0020] Jabbar, S. , J. Drury , J. N. Fordham , H. K. Datta , R. M. Francis , and S. P. Tuck . 2011 Osteoprotegerin, RANKL and bone turnover in postmenopausal osteoporosis. J. Clin. Pathol. 64:354–357. 21307155 10.1136/jcp.2010.086595

[phy213263-bib-0021] Jee, W. S. , and W. Yao . 2001 Overview: animal models of osteopenia and osteoporosis. J. Musculoskelet. Neuronal Interact. 1:193–207. 15758493

[phy213263-bib-0022] Jilka, R. L. , G. Hangoc , G. Girasole , G. Passeri , D. C. Williams , J. S. Abrams , et al. 1992 Increased osteoclast development after estrogen loss: mediation by interleukin‐6. Science (New York, N.Y.) 257:88–91. 10.1126/science.1621100 1621100

[phy213263-bib-0023] Khosla, S. , L. J. Melton , and B. L. Riggs . 2011 The unitary model for estrogen deficiency and the pathogenesis of osteoporosis: Is a revision needed? J. Bone Miner. Res. 26:441–451. 20928874 10.1002/jbmr.262 PMC3179298

[phy213263-bib-0024] Kovats, S. 2015 Estrogen receptors regulate innate immune cells and signaling pathways. Cell. Immunol.. Elsevier Inc., 294:63–69. 25682174 10.1016/j.cellimm.2015.01.018 PMC4380804

[phy213263-bib-0025] Lambert, K. C. , E. M. Curran , B. M. Judy , D. B. Lubahn , and D. M. Estes . 2004 Estrogen receptor‐alpha deficiency promotes increased TNF‐alpha secretion and bacterial killing by murine macrophages in response to microbial stimuli in vitro. J. Leukoc. Biol. 75:1166–1172. 15020652 10.1189/jlb.1103589

[phy213263-bib-0026] Laukoetter, M. G. , P. Nava , W. Y. Lee , E. A. Severson , C. T. Capaldo , B. A. Babbin , et al. 2007 JAM‐A regulates permeability and inflammation in the intestine in vivo. J. Exp. Med. 204:3067–3076. 18039951 10.1084/jem.20071416 PMC2150975

[phy213263-bib-0027] Lee, S. H. 2015 Intestinal permeability regulation by tight junction: implication on inflammatory bowel diseases. Intest. Res. 13:11. 25691839 10.5217/ir.2015.13.1.11 PMC4316216

[phy213263-bib-0028] Lee, J. S. S. , C. M. M. Tato , B. Joyce‐Shaikh , F. Gulan , C. Cayatte , Y. Chen , et al. 2015 Interleukin‐23‐Independent IL‐17 Production Regulates Intestinal Epithelial Permeability. Immunity 43:1–12. 26431948 10.1016/j.immuni.2015.09.003 PMC6044435

[phy213263-bib-0029] Lélu, K. , S. Laffont , L. Delpy , P.‐E. Paulet , T. Périnat , S. A. Tschanz , et al. 2011 Estrogen Receptor {alpha} Signaling in T Lymphocytes Is Required for Estradiol‐Mediated Inhibition of Th1 and Th17 Cell Differentiation and Protection against Experimental Autoimmune Encephalomyelitis. J. Immunol. 187:2386–2393. 21810607 10.4049/jimmunol.1101578

[phy213263-bib-0030] Li, X. , M. Stankovic , B. P. L. Lee , M. Aurrand‐Lions , C. N. Hahn , Y. Lu , et al. 2009 JAM‐C induces endothelial cell permeability through its association and regulation of *β*3 integrins. Arterioscler. Thromb. Vasc. Biol. 29:1200–1206. 19461049 10.1161/ATVBAHA.109.189217

[phy213263-bib-0031] Li, J.‐Y. , B. Chassaing , A. M. Tyagi , C. Vaccaro , T. Luo , J. Adams , et al. 2016 Sex steroid deficiency‐associated bone loss is microbiota dependent and prevented by probiotics. J. Clin. Investig. American Society for Clinical Investigation, 126:2049–2063. 27111232 10.1172/JCI86062 PMC4887186

[phy213263-bib-0032] Lin, F.‐H. , J. B. Chang , M. H. McGuire , J. A. Yee , and B. E. Brigman . 2010 Biphasic effects of interleukin‐1beta on osteoblast differentiation in vitro. J. Orthop. Res. 28:958–964. 20108347 10.1002/jor.21099

[phy213263-bib-0033] Lisabeth, L. , and C. Bushnell . 2012 Stroke risk in women: The role of menopause and hormone therapy. Lancet Neurol. 11:82–91. 22172623 10.1016/S1474-4422(11)70269-1 PMC3615462

[phy213263-bib-0034] Looijer‐van Langen, M. , N. Hotte , L. A. Dieleman , E. Albert , C. Mulder , and K. L. Madsen . 2011 Estrogen receptor‐*β* signaling modulates epithelial barrier function. Am. J. Physiol. Gastrointest. Liver physiol. 300:G621–G626. 21252046 10.1152/ajpgi.00274.2010

[phy213263-bib-0035] Ma, T. Y. , M. A. Boivin , D. Ye , A. Pedram , and H. M. Said . 2005 Mechanism of TNF‐{alpha} modulation of Caco‐2 intestinal epithelial tight junction barrier: role of myosin light‐chain kinase protein expression. Am. J. Physiol. Gastrointest. Liver Physiol. 288:G422–G430. 15701621 10.1152/ajpgi.00412.2004

[phy213263-bib-0036] Malutan, A. M. , M. Dan , C. Nicolae , and M. Carmen . 2014 Proinflammatory and anti‐inflammatory cytokine changes related to menopause. Menopausal Review 3:162–168. 10.5114/pm.2014.43818 PMC4520358 26327849

[phy213263-bib-0037] Merz, E. , D. Miric‐Tesanic , F. Bahlmann , G. Weber , and S. Wellek . 1996 Sonographic size of uterus and ovaries in pre‐ and postmenopausal women. Ultrasound Obstet. Gynecol. 7:38–42. 8932630 10.1046/j.1469-0705.1996.07010038.x

[phy213263-bib-0038] Neunlist, M. , L. Van Landeghem , M. M. Mahé , P. Derkinderen , S. B. des Varannes , and M. Rolli‐Derkinderen . 2013 The digestive neuronal‐glial‐epithelial unit: a new actor in gut health and disease. Nat. Rev. Gastroenterol. Hepatol. 10:90–100. 23165236 10.1038/nrgastro.2012.221

[phy213263-bib-0039] Neurath, M. F. 2014 Cytokines in inflammatory bowel disease'., Nature reviews. Immunology. Nature Publishing. Group 14:329–342. 10.1038/nri3661 24751956

[phy213263-bib-0040] Ohlsson, C. , C. Engdahl , F. Fåk , A. Andersson , S. H. Windahl , H. H. Farman , et al. 2014 Probiotics protect mice from ovariectomy‐induced cortical bone loss. PLoS ONE 9:e92368. 24637895 10.1371/journal.pone.0092368 PMC3956931

[phy213263-bib-0041] Orlova, V. V , M. Economopoulou , F. Lupu , S. Santoso , and T. Chavakis . 2006 Junctional adhesion molecule‐C regulates vascular endothelial permeability by modulating VE‐cadherin‐mediated cell‐cell contacts. J.Exp.Med 203:2703–2714. 17116731 10.1084/jem.20051730 PMC2118160

[phy213263-bib-0042] Pacifici, R. , J. L. Vannice , L. Rifas , and R. B. Kimble . 1993 Monocytic secretion of interleukin‐1 receptor antagonist in normal and osteoporotic women: effects of menopause and estrogen/progesterone therapy. J. Clin. Endocrinol. Metab. 77:1135–1141. 8077304 10.1210/jcem.77.5.8077304

[phy213263-bib-0043] Pfeilschifter, J. , R. Köditz , M. Pfohl , and H. Schatz . 2002 Changes in proinflammatory cytokine activity after menopause. Endocr. Rev. 23:90–119. 11844745 10.1210/edrv.23.1.0456

[phy213263-bib-0044] Phiel, K. L. , R. A. Henderson , S. J. Adelman , and M. M. Elloso . 2005 Differential estrogen receptor gene expression in human peripheral blood mononuclear cell populations. Immunol. Lett. 97:107–113. 15626482 10.1016/j.imlet.2004.10.007

[phy213263-bib-0045] Pinchuk, L. M. , and N. M. Filipov . 2008 Differential effects of age on circulating and splenic leukocyte populations in C57BL/6 and BALB/c male mice. Immun. Ageing 5:1. 18267021 10.1186/1742-4933-5-1 PMC2268915

[phy213263-bib-0046] Portman, D. J. , and M. L. S. Gass . 2014 Genitourinary syndrome of menopause: new terminology for vulvovaginal atrophy from the international society for the study of women's sexual health and the North American menopause society. J. Sex. Med. 11:2865–2872. 25155380 10.1111/jsm.12686

[phy213263-bib-0047] Strober, W. , and I. J. Fuss . 2011 Proinflammatory cytokines in the pathogenesis of inflammatory bowel diseases. Gastroenterology Elsevier Inc 140:1756–1767. 21530742 10.1053/j.gastro.2011.02.016 PMC3773507

[phy213263-bib-0048] Suzuki, T. 2013 Regulation of intestinal epithelial permeability by tight junctions. Cell. Mol. Life Sci. 70:631–659. 22782113 10.1007/s00018-012-1070-x PMC11113843

[phy213263-bib-0049] Turner, J. R. 2009 Intestinal mucosal barrier function in health and disease. Nat. Rev. Immunol. 9:799–809. 19855405 10.1038/nri2653

[phy213263-bib-0050] Ulluwishewa, D. , R. C. Anderson , W. C. McNabb , P. J. Moughan , J. M. Wells , and N. C. Roy . 2011 Regulation of tight junction permeability by intestinal bacteria and dietary components. J. Nutr. 141:769–776. 21430248 10.3945/jn.110.135657

[phy213263-bib-0051] Wada‐Hiraike, O. , O. Imamov , H. Hiraike , K. Hultenby , T. Schwend , Y. Omoto , et al. 2006 Role of estrogen receptor beta in colonic epithelium. Proc. Natl Acad. Sci. USA 103:2959–2964. 16477031 10.1073/pnas.0511271103 PMC1413854

[phy213263-bib-0052] Walkin, L. , S. E. Herrick , A. Summers , P. E. Brenchley , C. M. Hoff , R. Korstanje , et al. 2013 The role of mouse strain differences in the susceptibility to fibrosis: a systematic review. Fibrogenesis Tissue Repair 6:18. 24294831 10.1186/1755-1536-6-18 PMC3849643

[phy213263-bib-0053] Watanabe, H. , K. Numata , T. Ito , K. Takagi , and A. Matsukawa . 2004 Innate Immune Response in Th1‐ and Th2‐Dominant Mouse Strains. Shock 22:460–466. 15489639 10.1097/01.shk.0000142249.08135.e9

[phy213263-bib-0054] Weber, M. T. , P. M. Maki , and M. P. McDermott . 2014 Cognition and mood in perimenopause: a systematic review and meta‐analysis. J. Steroid Biochem. Mol. Biol. 142:90–98. 23770320 10.1016/j.jsbmb.2013.06.001 PMC3830624

